# ID2 secures cDC1 specification by antagonizing E proteins at a pleiotropic *Zeb2* enhancer

**DOI:** 10.21203/rs.3.rs-7455813/v1

**Published:** 2025-09-04

**Authors:** Feiya Ou, Tian-Tian Liu, Siling Du, Jing Chen, Alyssa R. Koch, Magdalena Kraft, Theresa L. Murphy, Kenneth M. Murphy

**Affiliations:** 1 Department of Pathology and Immunology, Washington University in St. Louis School of Medicine, St. Louis, MO, USA;; 2 Present address: Department of Immunology and Microbiology, The Scripps Research Institute, La Jolla, CA, USA;; 3 Brain Immunology and Glia (BIG) Center, Washington University in St. Louis School of Medicine, St. Louis, MO, USA;; 4 Medical Scientist Training Program, Washington University in St. Louis School of Medicine, St. Louis, MO, USA

## Abstract

The transcriptional regulator ID2 is required for type 1 classical dendritic cell (cDC1) specification, yet the mechanism has remained obscure. We previously identified the *Zeb2* −165-kb enhancer as key to normal hematopoiesis, controlled by competing CEBP and NFIL3 inputs during myeloid dendritic cell divergence. Here, we uncover an unprecedented role for E proteins in myelopoiesis and demonstrate that ID2 promotes cDC1 development by antagonizing E protein activity at E-boxes within the *Zeb2* enhancer. Deleting these E-boxes abolishes lymphoid B cell and plasmacytoid dendritic cell (pDC) development while skewing myelopoiesis toward cDC1s. Remarkably, E-box deletion rescues cDC1 development in *Id2*-deficient mice. These findings support a two-step model in which NFIL3 transiently represses *Zeb2*, followed by ID2-mediated inhibition of E proteins to stabilize cDC1 fate specification. Further, this work defines a paradigm of “site-specific pleiotropy,” wherein distinct transcription factor motifs–E-boxes and CEBP sites–within a single enhancer direct diverse cell fates.

## INTRODUCTION

Zinc finger E-box-binding homeobox 2 (ZEB2) is a transcriptional repressor that plays crucial roles in embryonic and adult hematopoiesis. Notably, ZEB2 is required for embryonic hematopoietic stem cell (HSC) differentiation, and *Zeb2* deletion impairs the supply of angiogenic factors from circulating hematopoietic cells, leading to cephalic hemorrhage, vascular instability, and embryonic lethality^1^. ZEB2 is also critical in maintaining the identity of tissue-resident macrophages, such as microglia and Kupffer cells, which are derived from embryonic precursors^2^.

In adult hematopoiesis, ZEB2 regulates the development of diverse lymphoid and myeloid lineages. Among lymphoid cells, ZEB2 is essential for B cell development^3,4^, natural killer (NK) cell maturation^5^, and the generation of terminal effector CD8 T (Teff) cells during infection^6,7^. Among myeloid lineages, ZEB2 promotes monocytes and type 2 classical dendritic cell (cDC2) development while suppressing type 1 classical dendritic cell (cDC1) development^3,8–10^. ZEB2 is also indispensable for the development of plasmacytoid dendritic cells (pDCs)^11,12^, which arise from both lymphoid and myeloid progenitors^13^.

The requirements for the transcriptional repressor nuclear factor interleukin-3-regulated protein (NFIL3) and the E protein antagonist inhibitor of DNA binding 2 (ID2) in cDC1 development have been known for some time^14–16^. A previous study from our lab, using epistasis analysis, demonstrated that both *Nfil3* and *Id2* promote cDC1 development by repressing *Zeb2* expression^9^. While a direct role in suppressing *Zeb2* has been ascribed to NFIL3, the precise molecular mechanism for the requirement of ID2 has remained elusive.

We previously identified an enhancer located 165 kb upstream of the *Zeb2* transcription start site that is essential for *Zeb2* expression in the adult hematopoietic system. Deletion of this *Zeb2* −165-kb enhancer (Δ−165 mice) led to a near-complete loss of B cells, absence of pDCs and monocytes, impaired NK cell maturation, and defective Teff cell differentiation^4^–phenotypes mirroring *Zeb2* ablation using various Cre-loxP-based approaches. More recently, we showed that disruption of three CCAAT-Enhancer-Binding Protein (CEBP) motifs within the enhancer in Δ1+2+3 mice^10^ (hereafter referred to as ΔC mice) selectively impairs monocytes and cDC2s, while lymphoid cells remain largely unaffected. The phenotypic differences between Δ−165 and ΔC mice suggest that TF binding sites (TFBSs) other than CEBP sites mediate *Zeb2* −165-kb enhancer activity in lymphoid lineages.

In this study, we identify E-boxes within the *Zeb2* −165-kb enhancer as key regulators of lymphoid-specific enhancer activity, crucial for normal lymphopoiesis. We further demonstrate that both E-boxes and CEBP sites facilitate enhancer activity in myeloid lineages. This configuration enables the enhancer to integrate distinct inputs and has critical implications for classical dendritic cell (cDC) lineage divergence: we show that NFIL3 and ID2 repress *Zeb2* −165-kb enhancer activity in a stepwise manner by displacing CEBPs and E proteins, respectively–a process required for cDC1 specification.

Beyond elucidating a mechanism of *Zeb2* regulation, our findings establish the *Zeb2* −165-kb enhancer as a new paradigm of enhancer pleiotropy. Pleiotropic enhancers are defined by their ability to control gene expression across multiple biological contexts^17,18^. We demonstrate that the *Zeb2* −165-kb enhancer exerts pleiotropic control over lymphoid and myeloid lineages through separate TF motifs–E-boxes in lymphoid cells, and a combination of CEBP sites and E-boxes in myeloid cells. Thus, we propose a mechanistic model of “site-specific pleiotropy”, in which lineage-restricted transcription is regulated by discrete TF motifs within a single enhancer, rather than by shared TFBSs reused across multiple cell types.

## RESULTS

### E proteins and CEBPs bind the *Zeb2* −165-kb enhancer

We previously observed that deletion of the *Zeb2* −165-kb enhancer results in broad defects across both lymphoid and myeloid lineages^4^. In contrast, targeted mutation of CEBP sites within this enhancer leads only to myeloid-specific defects^10^. To account for this difference, we hypothesized that additional TFBSs within the same enhancer may be responsible for its activity in lymphoid lineages.

Notably, we had identified three conserved E-boxes (named E2, E3, and E4) within this enhancer, which are bound by E proteins such as E2A and E2–2 *in vitro*^4^. By reanalyzing publicly available ChIP-seq and CUT&RUN datasets, we confirmed that in addition to CEBPs, E proteins such as E2A and HEB also bind the *Zeb2* −165-kb enhancer in primary cells and cell lines (Figures S1A and 1A). E2A binding was observed in pro-B and pre-B cells, suggesting that the enhancer is a bona fide target of E2A *in vivo*, consistent with the requirement of both E2A and ZEB2 in B cell development^3,19–22^. This regulatory logic appears conserved in the human orthologous region, where CEBPB, E2A, E2–2, and HEB all bind the syntenic enhancer in human cell lines (Figure S1B).

To further investigate the relevance of E protein binding, we examined their expression patterns in hematopoietic progenitors. Reanalysis of publicly available scRNA-seq and bulk RNA-seq datasets revealed that *Tcf3*, *Tcf4*, and *Tcf12* (encoding E2A, E2–2, and HEB, respectively) are expressed in pDCs, pre-pDCs, and pDC-like cells (Figures S2A and S2B), which have been associated with lymphoid origins^13,23–25^. Interestingly, myeloid progenitors such as monocyte-dendritic cell progenitors (MDPs) and common dendritic cell progenitors (CDPs) also expressed these genes to some degree (Figures S2B and S2C).

**Figure 1.**
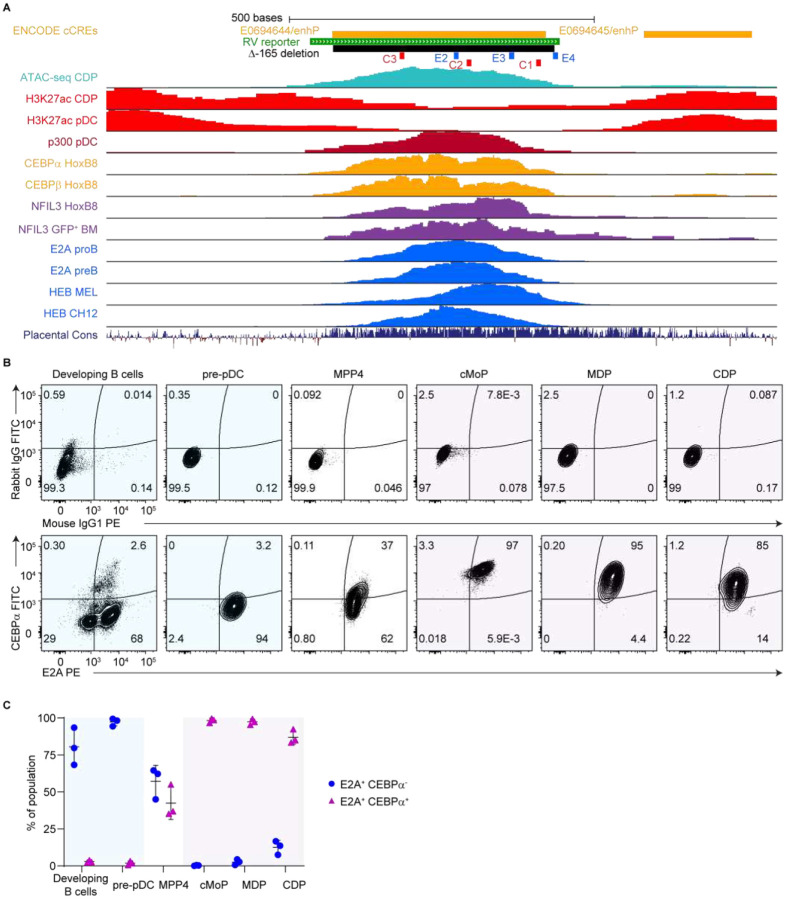
Both CEBPs and E proteins bind the *Zeb2* −165-kb enhancer. (A) Genomic features surrounding the *Zeb2* −165-kb enhancer (chr2:45,279,107–45,280,206; mm10). Locations of the retroviral (RV) reporter, the Δ−165 deletion, CEBP sites (C1, C2, and C3), and E-boxes (E2, E3, and E4) are shown. ENCODE candidate *cis*-regulatory elements (cCREs), ATAC-seq, ChIP-seq, CUT&RUN, and placental mammal base-wise conservation (Placental Cons) tracks are displayed for the indicated targets and cell types. Y-axes are auto-scaled. (B and C) Intracellular staining of WT C57BL/6 BM using isotype control or using E2A and CEBPα antibodies (n = 3 mice; three independent experiments). (B) Representative flow cytometry plots showing isotype control (top) and E2A/CEBPα (bottom) staining in the indicated populations. (C) Frequencies of E2A^+^ CEBPα^−^ and E2A^+^ CEBPα^+^ cells within the indicated populations (mean ± SD).

To validate these observations at the protein level, we performed intracellular staining for CEBPα and E2A across multiple bone marrow (BM) progenitor populations. Lymphoid populations, including developing B cells and pre-pDCs, exhibited E2A expression and lacked CEBPα (Figures 1B, 1C, S2D, and S2E). Multipotent progenitor 4 (MPP4), which retains both lymphoid and myeloid potentials^26,27^, contained an E2A^+^ single-positive fraction as well as an E2A^+^ CEBPα^+^ double-positive fraction. In contrast, myeloid progenitors such as common monocyte progenitors (cMoPs), MDPs, CDPs, and pre-cDC2s were predominantly double-positive for E2A and CEBPα (Figures 1B, 1C, S2D, and S2E).

In summary, the *Zeb2* −165-kb enhancer is bound by both CEBPs and E proteins in a lineage-restricted fashion. While lymphoid progenitors selectively express E2A, myeloid progenitors co-express E2A and CEBPα. These observations led us to revise our hypothesis: E proteins may contribute to enhancer activity not only in lymphoid cells but also in myeloid lineages.

### E-boxes within the *Zeb2* −165-kb enhancer are required for normal lymphopoiesis

**Figure 2.**
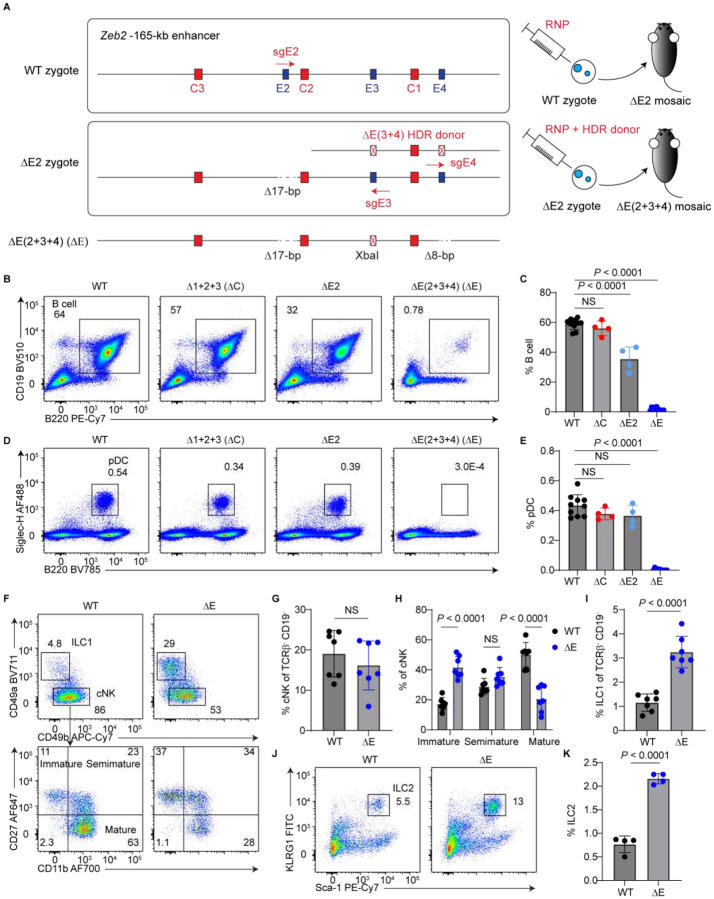
E-boxes within the *Zeb2* −165-kb enhancer are required for normal lymphopoiesis. (A) Schematic illustrating *Zeb2* −165-kb enhancer variants with indicated single guide RNAs (sgRNAs), the single-stranded homology-directed repair (HDR) donor, and specific E-box mutations. The ΔE(2+3+4) (ΔE) allele was generated through two rounds of CRISPR/Cas9 targeting, using the ΔE2 allele as an intermediate. All mice were on a C57BL/6 background. RNP, ribonucleoprotein. (B-I) Flow cytometric analysis of spleens from mice homozygous for the indicated *Zeb2* −165-kb enhancer alleles. (B) Representative plots showing B cell gating. (C) Frequencies of B cells among splenocytes (n = 4–11 mice; six independent experiments). (D) Representative plots showing pDC gating. (E) Frequencies of pDCs among splenocytes (n = 4–11 mice; six independent experiments). (F) Representative plots showing ILC1s and cNK cells (top), as well as cNK maturation stages (bottom). Pre-gate: TCRβ^−^ CD19^−^ cells. (G) Frequencies of cNK cells among TCRβ^−^ CD19^−^ cells (n = 7 mice; six independent experiments). (H) Frequencies of immature, semimature, and mature cNK subsets among total cNK cells (n = 7 mice; six independent experiments). (I) Frequencies of ILC1s among TCRβ^−^ CD19^−^ cells (n = 7 mice; six independent experiments). (J and K) Flow cytometric analysis of small intestine lamina propria (SILP) lymphocytes from WT and homozygous ΔE mice (n = 4 mice; two independent experiments). (J) Representative plots showing ILC2 gating. Pre-gate: CD45^+^ CD3ε^−^ CD19^−^ cells. (K) Frequencies of ILC2s among total SILP lymphocytes. Bar graphs represent mean ± SD. Statistical significance was assessed using one-way ANOVA or unpaired two-tailed t tests, as appropriate; NS, not significant.

To directly assess the physiological significance of E protein binding at the *Zeb2* −165-kb enhancer, we generated mouse strains lacking one or more E-boxes using CRISPR/Cas9. We first established a mouse line lacking E2 (ΔE2) (Figure 2A). Subsequently, we generated another line lacking E2, E3, and E4 by an additional round of CRISPR/Cas9 targeting on ΔE2 mice, resulting in the ΔE(2+3+4) strain (hereafter referred to as ΔE) (Figure 2A).

Since the ΔE variant of the *Zeb2* −165-kb enhancer retains intact CEBP sites (Figure S3A), we sought to rule out the possibility that the E-box mutations inadvertently disrupted CEBP binding by altering enhancer syntax or affecting pioneering activities. To address this, we conducted CEBPα CUT&RUN on BM lin^−^ KIT^int-lo^ FLT3^+^ cells from WT, ΔC, and ΔE mice, which encompass a range of hematopoietic progenitors. As anticipated, neither ΔC nor ΔE mice exhibited global changes in CEBPα binding across the genome (Figure S3B). Within the *Zeb2* −165-kb enhancer specifically, ΔC mice exhibited markedly reduced CEBPα binding, whereas ΔE mice showed no reduction of CEBPα binding compared to WT mice (Figure S3C). These results suggest that the ΔE allele provides a suitable model to isolate the functional role of E protein binding at the *Zeb2* −165-kb enhancer.

As reported previously^10^, ΔC mice retain a normal splenic B cell population (Figures 2B and 2C). In contrast, ΔE2 mice showed a ~2-fold reduction in B cell frequency, suggesting a partial requirement of E2 in B cell development. Strikingly, ΔE mice displayed a near-complete loss of B cells (Figures 2B and 2C), indicating that the E-boxes within the *Zeb2* −165-kb enhancer are indispensable for B cell development. This observation aligns with E2A binding at the enhancer in pro-B and pre-B cells (Figure 1A). ΔE mice also showed a complete absence of pDCs (Figures 2D and 2E), suggesting that E-boxes are required for both lymphoid- and myeloid-derived pDC development. Additionally, ΔE mice exhibited impaired maturation of conventional NK (cNK) cells, accompanied by an accumulation of immature cNK cells (Figures 2F–2H).

Interestingly, ZEB2-independent innate lymphoid cells (ILCs), such as ILC1s and ILC2s, were significantly increased in ΔE mice (Figures 2F, 2I–2K). In agreement with these peripheral alterations, BM ILC progenitors such as CHILPs and ILC2Ps were markedly expanded in ΔE mice (Figures S4A–S4D). These findings suggest that E protein-mediated enhancer activity normally promotes B cell and pDC specification while suppressing alternative ILC lineages. In summary, E-boxes in the *Zeb2* −165-kb enhancer are crucial for normal lymphopoiesis by enabling B cell and pDC development, supporting cNK cell maturation, and limiting ILC1 and ILC2 numbers.

### Both E-boxes and CEBP sites in the *Zeb2* −165-kb enhancer are required for normal myelopoiesis

Since E proteins are expressed in both lymphoid and myeloid progenitors (Figures 1B and 1C), we next examined the myeloid compartment in ΔE mice. BM from ΔE mice exhibited reduced frequencies of cMoPs and Ly-6C^+^ monocytes compared to WT controls, though these reductions were less severe than those observed in ΔC mice (Figures S4E–S4G). In contrast, frequencies of Ly-6C^−^ monocytes in the BM and total splenic monocytes in ΔE mice remained comparable to WT (Figures S4H–S4J). These findings suggest that high levels of CEBP expression in monocytes may compensate for the absence of E-boxes, maintaining enhancer activity in this lineage.

**Figure 3.**
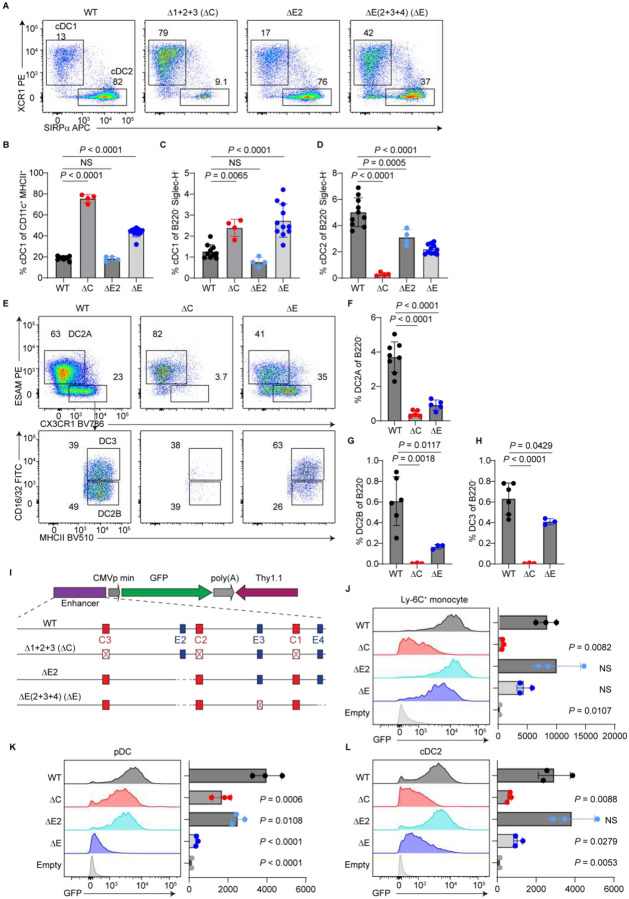
Both CEBP sites and E-boxes within the *Zeb2* −165-kb enhancer regulate cDC subset differentiation. (A-H) Flow cytometric analysis of spleens from mice homozygous for the indicated *Zeb2* −165-kb enhancer alleles. (A) Representative plots showing cDC1 and cDC2 gating (n = 4–11 mice; six independent experiments). Pre-gate: B220^−^ Siglec-H^−^ CD11c^+^ MHCII^+^ cells (cDCs). (B) Frequencies of cDC1s among cDCs from experiments in (A). (C) Frequencies of cDC1s among B220^−^ Siglec-H^−^ cells from experiments in (A). (D) Frequencies of cDC2s among B220^−^ Siglec-H^−^ cells from experiments in (A). (E) Representative plots showing cDC2 subset gating (n = 3–8 mice; four independent experiments). Pre-gate: B220^−^ CD11c^+^ MHCII^+^ SIRPα^+^ cells (cDC2s). (F) Frequencies of DC2As among B220^−^ cells from experiments in (E). (G) Frequencies of DC2Bs among B220^−^ cells from experiments in (E). (H) Frequencies of DC3s among B220^−^ cells from experiments in (E). (I) Schematic of RV reporter constructs in which GFP expression is driven by distinct *Zeb2* −165-kb enhancer variants, used in experiments in (J-L). (J) WT C57BL/6 BM cells cultured in KIT, IL-3, and IL-6 were spin-infected with RV reporters on day 1 of culture and analyzed by flow cytometry on day 3 (n = 2–3 mice; two independent experiments). Shown are representative GFP histogram in transduced Ly-6C^+^ monocytes (left) and GFP gMFI (right). Pre-gated on Thy1.1+ KIT^−^ M-CSFR^−^ CD11b+ Ly6C+ cells. (K and L) WT C57BL/6 BM cells were cultured in a two-step KIT/FLT3L protocol to generate DCs. RV reporters were introduced by spin-infection on day 1 of culture. GFP expression was analyzed by flow cytometry on day 8 (n = 2–3 mice; two independent experiments). (K) Representative GFP histogram in transduced pDCs (left) and GFP gMFI (right). Pre-gate: Thy1.1^+^ B220^+^ Siglec-H^+^ cells. (L) Representative GFP histogram in transduced cDC2s (left) and GFP gMFI (right). Pre-gate: Thy1.1^+^ B220^−^ Siglec-H^−^ CD11c^+^ MHCII^+^ SIRPα^+^ cells. Bar graphs represent mean ± SD. Statistical significance was assessed using one-way ANOVA; NS, not significant.

We next assessed cDC development. Like ΔC mice, ΔE mice exhibited a substantial expansion of lineage-committed pre-cDC1s in the BM and increased frequencies of differentiated cDC1s in the spleen (Figures S5A, S5B, 3A–3C). ΔE mice also showed reduced frequencies of pre-cDC2s, a myeloid cDC2 progenitor, though the reduction was milder than in ΔC mice (Figures S5C and S5D). Strikingly, both ΔE and ΔC mice completely lacked CD11c^lo^ and CD11c^hi^ transitional DCs (tDC^lo^ and tDC^hi^) (Figures S5E–S5G), which are cDC2 progenitors believed to arise from a lymphoid origin^25^. This observation adds to the profound lymphoid deficiencies in ΔE mice. In line with these upstream defects, ΔE mice displayed a significant reduction in splenic cDC2s (Figures 3A and 3D). Further subset analysis revealed that all three cDC2 subsets were reduced in ΔE mice: DC2As, DC2Bs, and DC3s (Figures 3E–3H). In comparison, ΔC mice showed a more severe cDC2 deficiency (Figures 3A and 3D), retaining only a small fraction of DC2As and completely lacking DC2Bs and DC3s (Figures 3E–3H). These data suggest that *Zeb2* −165-kb enhancer activity in myeloid DC progenitors is jointly supported by E-boxes and CEBP-binding sites—both required to balance cDC1 versus cDC2 lineage output.

To directly visualize enhancer activity across lineages, we generated retroviral (RV) GFP reporters driven by specific *Zeb2* −165-kb enhancer variants and transduced them into BM cells cultured under lineage-appropriate differentiation conditions (Figure 3I). In culture-derived monocytes, only the ΔC variant showed a lack of enhancer activity (Figure 3J). In contrast, in pDCs, all three enhancer variants (ΔC, ΔE2, and ΔE) exhibited decreased activities to varying degrees, with the ΔE variant appearing completely inactive (Figure 3K). In cDC2s, both ΔC and ΔE variants displayed diminished activities (Figure 3L). Together with the *in vivo* phenotypes, these results suggest that monocytes primarily rely on CEBP for enhancer activity, pDCs are predominantly dependent on E-boxes, and cDC2s require both CEBP sites and E-boxes for proper development.

### Both E-boxes and CEBP sites within the *Zeb2* −165-kb enhancer are required for normal *Zeb2* expression

**Figure 4.**
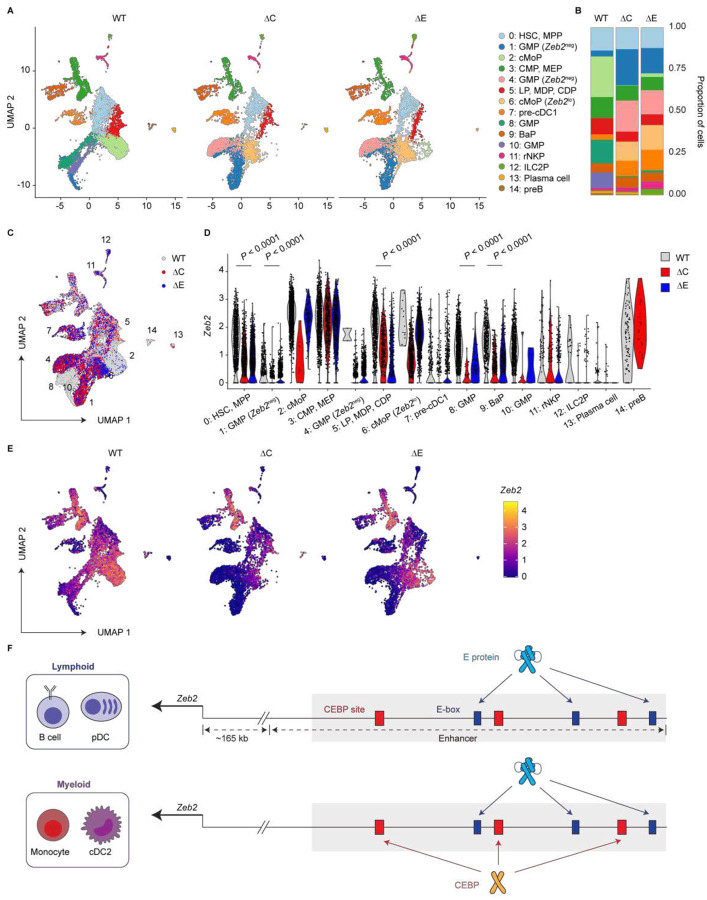
Normal *Zeb2* expression requires both CEBP sites and E-boxes in the −165-kb enhancer. (A-E) scRNA-seq analysis of sort-purified BM lin^−^ KIT^hi-int^ cells from mice homozygous for the indicated *Zeb2* −165-kb enhancer alleles (n = 3 mice per genotype). Lineage (lin) markers used for depletion included CD3ε, CD8β, CD11b, CD19, B220, Ly-6G, and TER-119. (A) UMAP of cells split by genotype. (B) Stacked bar plot showing the proportions of each identified cell type for the indicated genotypes. Color scheme matches (A). (C) UMAP from (A), now grouped by genotype. (D) Violin plot showing *Zeb2* expression across identified cell types for each genotype. (E) *Zeb2* expression projected onto the UMAP shown in (A). (F) Schematic summarizing TF activities at *Zeb2* −165-kb enhancer in the indicated lymphoid and myeloid cell types. Statistical significance was assessed using two-tailed Wilcoxon rank sum tests.

To directly assess the contributions of CEBP sites and E-boxes to *Zeb2* expression across hematopoietic progenitor populations, we performed scRNA-seq on BM lin^−^ KIT^hi-int^ cells from WT, ΔC, and ΔE mice. Compared to WT, both ΔC and ΔE samples exhibited altered clustering patterns, reflecting changes in cellular composition (Figures 4A–4C and S6A). In line with the loss of splenic B cells observed by flow cytometry, ΔE mice completely lacked pre-B cells (cluster 14) and showed reduced plasma cells (cluster 13) (Figures 4A and 4B). ΔE mice also display an expansion of ILC2Ps (cluster 12) (Figures 4A and 4B), validating our prior flow cytometry results.

In addition to these lymphoid alterations, both ΔC and ΔE BM demonstrated marked shifts in myeloid progenitor populations. The frequency of pre-cDC1s (cluster 7) was increased in both variants (Figures 4A and 4B), consistent with flow cytometry results. Interestingly, granulocyte-monocyte progenitor (GMP) and cMoP clusters shifted positions in the UMAP space in both ΔC and ΔE samples relative to WT (Figures 4A and 4C). While WT GMPs (clusters 8 and 10) and cMoPs (cluster 2) expressed *Zeb2*, their ΔC and ΔE counterparts (clusters 1 and 4 for GMP and cluster 6 for cMoP) showed diminished or absent *Zeb2* expression (Figures 4D, 4E, and S6B). This suggests that both CEBP sites and E-boxes are indispensable for the normal *Zeb2* level required for the transcriptional identities of these myeloid progenitors. Beyond GMPs and cMoPs, *Zeb2* expression was reduced in several other populations in both ΔC and ΔE BM. These include cluster 0, encompassing hematopoietic stem cells (HSCs) and MPPs, cluster 5, which includes LPs, MDPs, and CDPs, and cluster 9, corresponding to basophil progenitors (BaPs) (Figures 4D and 4E).

These data suggest that distinct TF inputs mediate enhancer activity in a context-specific manner: HSCs, MPPs, and various myeloid progenitors use both CEBP sites and E-boxes for *Zeb2* expression, whereas lymphoid progenitors primarily depend on E-boxes. Together with flow cytometry evidence, these results further establish that distinct sets of motifs in different developmental and cellular contexts preferentially activate the pleiotropic *Zeb2* −165-kb enhancer (Figure 4F).

### NFIL3 promotes cDC1 specification through repressing *Zeb2*

**Figure 5.**
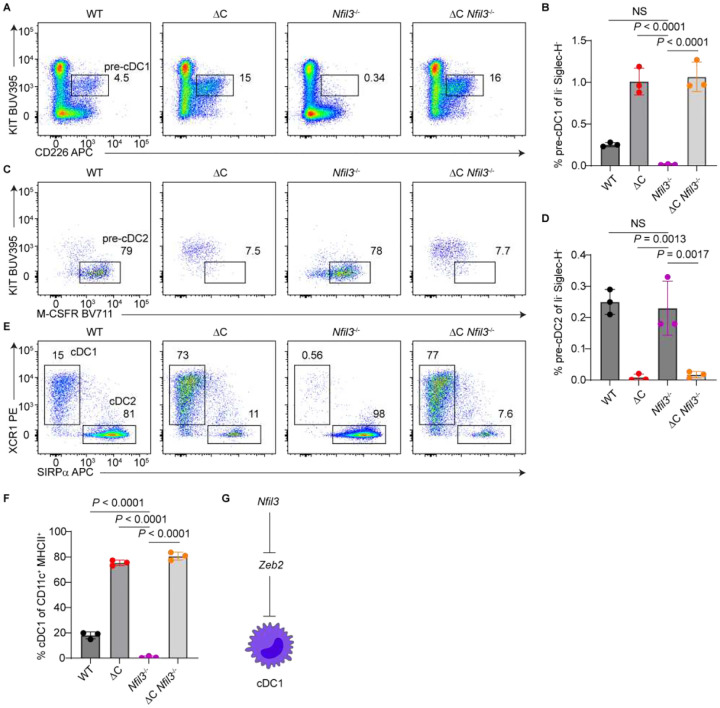
NFIL3 induces cDC1 fate specification through repressing *Zeb2*. (A-D) Flow cytometric analysis of BM from mice with the indicated genotypes (n = 3 mice; two independent experiments). ΔC refers to the homozygous *Zeb2* −165-kb enhancer ΔC allele (used consistently throughout the manuscript). (A) Representative plots showing pre-cDC1 gating. Pre-gate: lin^−^ Siglec-H^−^ FLT3^+^ cells. Lineage markers included CD3ε, CD19, B220, CD105, IL-7Rα, Ly-6G, and TER-119. (B) Frequencies of pre-cDC1s among lin^−^ Siglec-H^−^ cells. (C) Representative plots showing pre-cDC2 gating. Pre-gate: lin^−^ Siglec-H^−^ FLT3^+^ CD11c^−^ MHCII^−^ cells. Lineage markers as in (A). (D) Frequencies of pre-cDC2s among lin^−^ Siglec-H^−^ cells. (E and F) Flow cytometric analysis of spleens from mice with the indicated genotypes (n = 3 mice; two independent experiments). (E) Representative plots showing cDC1 and cDC2 populations. Pre-gate: B220^−^ Siglec-H^−^ CD11c^+^ MHCII^+^ cells (cDCs). (F) Frequencies of cDC1s among cDCs. (G) Schematic summarizing the epistatic relationship between *Nfil3* and *Zeb2* in regulating cDC1 development. Bar graphs represent mean ± SD. Statistical significance was assessed using one-way ANOVA; NS, not significant.

Through epistasis experiments, we previously established that *Zeb2* functions downstream of *Nfil3* in the regulation of cDC1 specification^9^. More recently, we demonstrated that NFIL3 acts as a transcriptional repressor to promote cDC1 development and directly binds to the *Zeb2* −165-kb enhancer at CEBP sites^10^ (Figure 1A), suggesting a model in which NFIL3 promotes cDC1 lineage specification by antagonizing CEBP-mediated activation of the *Zeb2* −165-kb enhancer. To test this model genetically, we crossed *Nfil3*^−/−^ mice to ΔC mice, which lack CEBP binding at the *Zeb2* −165-kb enhancer (Figure S3C). As expected, *Nfil3*^−/−^ mice exhibited a complete absence of pre-cDC1s in the BM and cDC1s in the spleen. In contrast, ΔC *Nfil3*^−/−^ double mutants displayed increased frequencies of both pre-cDC1s and cDC1s, comparable to ΔC single mutants (Figures 5A–5F). The double mutants also showed similarly diminished pre-cDC2 and cDC2 populations as ΔC mice (Figures 5C–5E).

Given that ΔE mice also exhibit reduced *Zeb2* expression in myeloid progenitors (Figure 4D), we next crossed *Nfil3*^−/−^ mice to ΔE mice. Resembling the ΔC *Nfil3*^−/−^ phenotype, ΔE *Nfil3*^−/−^ display enhanced pre-cDC1 and cDC1 frequencies, indistinguishable from ΔE single mutants (Figures S7A–S7D). These results further confirm that NFIL3 promotes cDC1 specification exclusively through repression of *Zeb2* (Figure 5G), likely by inhibiting CEBP-mediated activation of the *Zeb2* −165-kb enhancer.

### NFIL3 promotes ILC1 and immature cNK cell development by repressing *Zeb2*

In addition to its role in cDC1 specification, NFIL3 is also required for the development of cNK cells and ILC1s^28,29^. To test whether *Zeb2* acts downstream of *Nfil3* in these lineages as well, we examined ΔE *Nfil3*^−/−^ double mutant mice. While *Nfil3*^−/−^ mice lack ILC1s and cNK cells, ΔE *Nfil3*^−/−^ mice developed both cell types (Figures S7E–S7G), although at somewhat reduced frequencies compared to ΔE single mutants. Further, cNK cells in ΔE *Nfil3*^−/−^ double mutant mice showed compromised maturation, consistent with the defect observed in ΔE single mutants (Figure S7E). These results suggest that NFIL3 supports the development of ILC1s and cNK cells at least in part by repressing *Zeb2* (Figure S7H).

### ID2 promotes cDC1 development through repressing *Zeb2*

**Figure 6.**
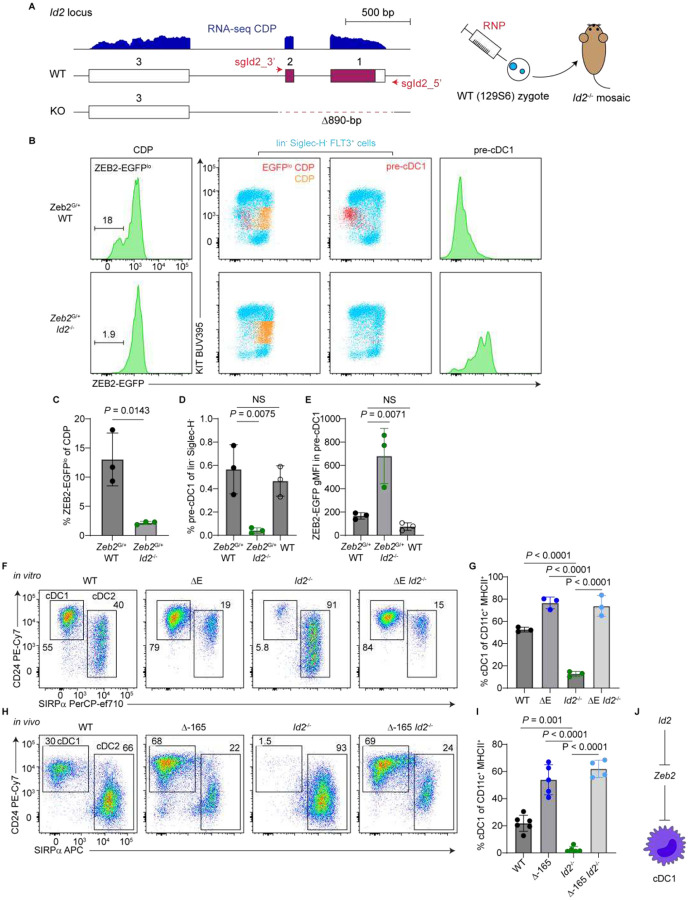
ID2 promotes cDC1 development by repressing *Zeb2*. (A) RNA-seq track of the *Id2* locus in CDPs and schematic illustrating the generation of the *Id2* knockout allele. sgRNAs and the resulting 890-bp deletion (chr12:25,095,282–25,096,171; mm10) are indicated. *Id2*^−/−^ mice were generated on a 129S6 background and maintained on a mixed 129S6.C57BL/6 background for experiments. (B-E) Flow cytometric analysis of ZEB2-EGFP expression in BM from mice with the indicated genotypes (n = 3 mice; three independent experiments). (B) Representative plots highlighting ZEB2-EGFP expression in the indicated populations. CDPs were gated as lin^−^ Siglec-H^−^ KIT^int^ FLT3^+^ M-CSFR^+^ CD11c^−^ MHCII^−^; pre-cDC1s were gated as lin^−^ Siglec-H^−^ KIT^int^ FLT3^+^ CD226^+^ cells. Lineage markers included CD3e, CD11b, CD19, B220, CD105, IL-7Ra, Ly-6G, and TER-119. (C) Frequencies of ZEB2-EGFP^lo^ CDPs among total CDPs. (D) Frequencies of pre-cDC1s among lin^−^ Siglec-H^−^ cells. (E) ZEB2-EGFP geometric mean fluorescence intensity (gMFI) in pre-cDC1s. (F and G) Flow cytometric analysis of *in vitro* KITL/FLT3L BM cultures from mice with the indicated genotypes (n = 3 mice; three independent experiments). ΔE refers to the homozygous *Zeb2* −165-kb enhancer ΔE allele (used consistently throughout the manuscript). (F) Representative plots showing cDC1 and cDC2 gating. Pre-gate: B220^−^ Siglec-H^−^ CD11c^+^ MHCII^+^ cells (cDCs). (G) Frequencies of cDC1s among cDCs. (H and I) Flow cytometric analysis of spleens from mice with the indicated genotypes (n = 4–6 mice; five independent experiments). Δ−165 refers to the homozygous *Zeb2* −165-kb enhancer deletion allele (used consistently throughout the manuscript). (H) Representative plots showing cDC1 and cDC2 gating. Pre-gate: B220^−^ Siglec-H^−^ CD11c^+^ MHCII^+^ cells (cDCs). (I) Frequencies of cDC1s among cDCs. (J) Schematic summarizing the epistatic relationship between *Id2* and *Zeb2* in cDC1 development. Bar graphs represent mean ± SD. Statistical significance was assessed using one-way ANOVA or unpaired two-tailed t tests, as appropriate; NS, not significant.

Like NFIL3, ID2 is a well-established positive regulator of cDC1 specification^9,15,16^. At the molecular level, ID2 is a helix-loop-helix protein lacking a basic DNA-binding domain and functions by antagonizing the activity of basic helix-loop-helix (bHLH) E proteins^30,31^. Having demonstrated that E-box-mediated activity of the *Zeb2* −165-kb enhancer suppresses cDC1 development (Figures 3A–3C), we hypothesized that ID2 supports cDC1 lineage specification by blocking E protein-mediated activation of the enhancer. To address this hypothesis, we first regenerated a new model of *Id2*^−/−^ mice. Because *Id2*^−/−^ mice are viable on a 129 background^32^ but not on a pure C57BL/6 background^33^, we deleted *Id2* on a 129S6 background (Figure 6A), and crossed into a mixed 129S6.C57BL/6 background for experiments.

To test whether ID2 represses *Zeb2* expression during cDC1 lineage specification, we crossed *Id2*^−/−^ mice to ZEB2-EGFP fusion protein reporter mice (*Zeb2*^G/+^). *Zeb2*^G/+^ CDPs exhibited a small subset of ZEB2-EGFP^lo^ cells (Figures 6B and 6C), previously identified as early cDC1 fate-specified cells upstream of pre-cDC1s^9^. By contrast, *Zeb2*^G/+^ *Id2*^−/−^ CDPs lacked this ZEB2-EGFP^lo^ population entirely, coinciding with a near complete absence of pre-cDC1s (Figures 6B–6D). Moreover, while *Zeb2*^G/+^ pre-cDC1s normally express low levels of ZEB2-EGFP, the few residual *Zeb2*^G/+^ *Id2*^−/−^ pre-cDC1s failed to repress ZEB2 expression (Figures 6B and 6E). Similar phenotypes were observed in *Zeb2*^G/G^ *Id2*^−/−^ mice carrying two copies of the *Zeb2*-EGFP allele (Figures S8A–D), further confirming that ID2 is required to downregulate ZEB2 during cDC1 lineage specification.

To test genetically whether ID2 supports cDC1 development by repressing *Zeb2*, we crossed *Id2*^−/−^ mice to ΔE mice. As expected, *Id2*^−/−^ mice completely lacked splenic cDC1s, while ΔE *Id2*^−/−^ double mutants showed a partial restoration of cDC1s, though this rescue did not reach statistical significance (Figures S8E and S8F). In contrast, ΔE *Id2*^−/−^ BM cultures showed a significant increase in cDC1 frequencies, markedly different from *Id2*^−/−^ cultures and phenocopying ΔE single mutants (Figures 6F and 6G).

Because the *in vivo* rescue was incomplete, we considered the possibility that cryptic E-boxes remained in the ΔE variant allowed for some E-protein-mediated *Zeb2* expression. We scanned the *Zeb2* −165-kb enhancer using Find Individual Motif Occurrences (FIMO) and identified six low-confidence E-box motifs in addition to the three high-confidence sites (E2, E3, and E4) deleted in the ΔE variant (Figure S8G). These additional sites may permit residual E protein binding in ΔE *Id2*^−/−^ mice, limiting the extent of cDC1 rescue. To eliminate this possibility, we crossed *Id2*^−/−^ mice to Δ−165 mice, which harbor a complete deletion of the *Zeb2* −165-kb enhancer (Figure S8G). Δ−165 *Id2*^−/−^ mice completely restored cDC1 development and exhibited elevated cDC1 frequencies, indistinguishable from Δ−165 single mutant mice (Figures 6H and 6I). Together, these results demonstrate that ID2 promotes cDC1 development through repressing *Zeb2* expression (Figure 6J), likely by blocking E protein-mediated activation of the *Zeb2* −165-kb enhancer.

### ID2 is dispensable for terminal cDC1 functions

Given the restored cDC1 development in Δ−165 *Id2*^−/−^ mice, this model provides a unique opportunity to examine the role of *Id2* in terminally differentiated cDC1s. We first examined the ability of Δ−165 *Id2*^−/−^ cDC1s to cross-present bacterial-associated antigen to naïve CD8 T cells. As anticipated, WT cDC1s, but not cDC2s, were able to cross-present heat-killed listeria-associated ovalbumin (HKLM-OVA) to naïve OT-I CD8 T cells, inducing their proliferation (Figures S9A and S9B). cDC1s from both Δ−165 single mutants and Δ−165 *Id2*^−/−^ double mutants induced OT-I proliferation levels comparable to WT cDC1s, suggesting that ID2 is dispensable for cDC1-mediated cross-presentation.

We then assessed the capacity of Δ−165 *Id2*^−/−^ cDC1s to produce IL-12 in response to *Toxoplasma gondii* soluble tachyzoite antigen (STAg). As expected, WT cDC1s but not cDC2s expressed both IL-12 p40 and IL-12 p35 upon STAg stimulation (Figures S9C and S9D). cDC1s from both Δ−165 single mutants and Δ−165 *Id2*^−/−^ double mutants exhibited similar levels of IL-12 expression, suggesting that ID2 is not required for this aspect of cDC1 function. Together, these data suggest that the cDC1s restored in Δ−165 *Id2*^−/−^ mice are functionally competent, and that ID2 is dispensable for the key effector functions of mature cDC1s, including antigen cross-presentation and IL-12 production.

### ID2 is required for Langerhans cell development independently of the *Zeb2* −165-kb enhancer

Langerhans cells (LCs) are skin-resident macrophages that share multiple characteristics with cDC1s, including the expression of several surface markers, the *Zbtb46* gene^12^, antigen presentation capacity^34^, and *Id2* dependence^16^. We asked whether the *Zeb2* −165-kb enhancer, which mediates ID2-dependent regulation in cDC1 development, also plays a role in restricting LC development. Immunofluorescence analysis of ear epidermal sheets revealed the presence of LCs in WT and Δ−165 mice, and their absence in *Id2*^−/−^ mice (Figure S9E). Notably, Δ−165 *Id2*^−/−^ mice also lacked LCs, indicating that *Id2* is required for LC development independently of the *Zeb2* −165-kb enhancer. This contrasts with cDC1s, in which enhancer deletion is sufficient to bypass the requirement for ID2.

### ZEB2 promotes B cell and pDC development by repressing ID2

**Figure 7.**
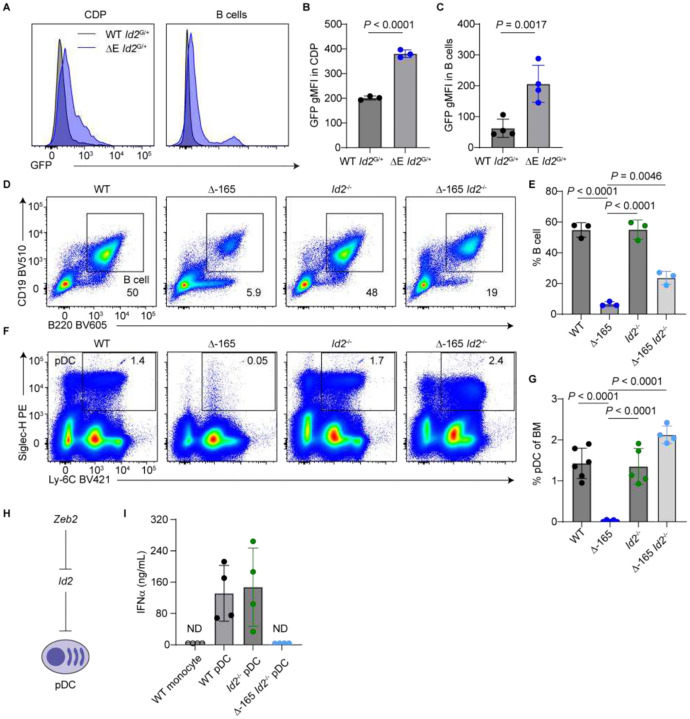
ZEB2 promotes B cell and pDC development by repressing ID2. (A-C) Flow cytometric analysis of *Id2*-GFP expression in BM CDPs and splenic B cells from WT and ΔE mice carrying an *Id2*-IRES-GFP reporter. CDPs were gated as lin^−^ Siglec-H^−^ KIT^int^ FLT3^+^ M-CSFR^+^ CD11c^−^ MHCII^−^ BM cells; B cells were gated as B220^+^ CD19^+^ splenocytes. (A) Representative histograms showing GFP expression in WT *Id2*^G/+^ (gray) and ΔE *Id2*^G/+^ (blue) cells. (B) GFP gMFI in CDPs (n = 3 mice; two independent experiments). (C) GFP gMFI in B cells (n = 4 mice; three independent experiments). (D and E) Flow cytometric analysis of splenic B cells from mice with the indicated genotypes (n = 3 mice; three independent experiments). (D) Representative plots showing B cell gating. (E) Frequencies of B cells among splenocytes. (F and G) Flow cytometric analysis of pDCs from BM of mice with the indicated genotypes. (F) Representative plots showing pDC gating in BM. (G) Frequencies of pDCs among BM cells (n = 4–6 mice; five independent experiments). (H) Schematic summarizing the epistatic relationship between *Zeb2* and *Id2* in pDC development. (I) IFN-α secretion analysis following CpG-A 2216 stimulation. BM monocytes (negative control) or pDCs were sort-purified from mice with the indicated genotypes and stimulated *in vitro* with CpG-A 2216 for 18 hours. Supernatants were analyzed for IFN-α levels by ELISA (n = 4 mice; four independent experiments). ND, not detected. Bar graphs represent mean ± SD. Statistical significance was assessed using one-way ANOVA or unpaired two-tailed t tests, as appropriate.

Next, we asked whether ZEB2 reciprocally promotes B cell and pDC development by repressing *Id2*. Prior studies have reported ectopic or elevated *Id2* expression in *Zeb2*-deficient contexts^4,9,11^, suggesting a mutual antagonism between these two factors^9^. We hypothesized ZEB2 facilitates B cell and pDC development through repressing *Id2* expression, thereby enabling E protein activity. To test this, we first crossed *Id2*-IRES-GFP reporter mice (*Id2*^G/+^) to ΔE mice, in which E-box-mediated *Zeb2* expression is impaired. Compared to WT *Id2*^G/+^ controls, ΔE *Id2*^G/+^ mice displayed increased GFP expression across multiple hematopoietic populations including CDPs (Figures 7A and 7B), DC2As, and CX3CR1^+^ DC2s encompassing DC2Bs and DC3s (Figures S10A–D). Notably, residual B cells in ΔE *Id2*^G/+^ mice also aberrantly expressed *Id2* (Figures 7A and 7C), consistent with the repressive role of ZEB2 on *Id2* expression.

To investigate the epistatic relationship between *Zeb2* and *Id2* in B cell and pDC development, we analyzed ΔE *Id2*^−/−^ double mutants. On a mixed 129S6.C57BL/6 background, ΔE mice displayed reduced B cell frequencies (Figures S10E and S10F), albeit less severe than on a pure C57BL/6 background. This reduction was partially rescued in ΔE *Id2*^−/−^ double mutants, suggesting that ZEB2 supports B cell development in part by repressing *Id2*. A similar trend was observed for pDCs: while ΔE mice completely lacked pDCs, ΔE *Id2*^−/−^ mice exhibited a partial restoration of this population (Figures S10G and S10H), supporting a parallel role for ZEB2 in pDC development through *Id2* repression.

To further validate this mechanism, we analyzed Δ−165 *Id2*^−/−^ double mutants, in which the *Zeb2* −165-kb enhancer is entirely deleted, leading to a more complete loss of *Zeb2* expression. Relative to Δ−165 single mutants, Δ−165 *Id2*^−/−^ mice showed increased frequencies of B cells (Figures 7D and 7E). Δ−165 *Id2*^−/−^ double mutants also exhibited a full rescue of pDCs in the BM and a partial rescue in the spleen (Figures 7F, 7G, S10I, and S10J). These epistasis experiments suggest that ZEB2 promotes B cell and pDC development, at least in part, by repressing *Id2* (Figure 7H).

Despite restoration of pDCs in ΔE *Id2*^−/−^ and Δ−165 *Id2*^−/−^ mice, these rescued pDCs did not fully phenocopy WT pDCs, as they exhibited reduced Siglec-H expression (Figures S10G, S10I, and 7F). To assess their functional competence, we measured IFN-α production following CpG-A 2216 stimulation. Whereas pDCs sort-purified from WT and *Id2*^−/−^ BM robustly secreted of IFN-α, Δ−165 *Id2*^−/−^ pDCs failed to do so (Figure 7I), indicating that ZEB2 is required not only for pDC development but also for their functional maturation.

## DISCUSSION

By uncovering the role of E proteins in myeloid development, we now explain the requirement for ID2 in cDC1 development. During cDC1 specification, transient suppression of the *Zeb2* −165-kb enhancer by NFIL3 is followed by permanent suppression mediated by the ID2-dependent blockade of E protein activity, locking the enhancer into an “off” state. We also define the *Zeb2* −165-kb enhancer as a pleiotropic regulatory element that integrates lineage-specific TF inputs to regulate *Zeb2* expression across diverse hematopoietic lineages. By analyzing ΔE and ΔC mice, we demonstrate that E-boxes and CEBP sites exhibit differential contributions across hematopoietic progenitors (Figure 4F). This study uncovers a novel paradigm of “site-specific enhancer pleiotropy”, wherein discrete TFBSs within a single enhancer drive lineage-specific gene expression.

Enhancer pleiotropy refers to a single enhancer controlling gene expression in multiple cell types. Prior studies often attribute this to “site pleiotropy”, where the same TFBSs are used across contexts^17,18,35^. Fewer studies have suggested the alternative model, in which different TFBSs activate the same enhancer in distinct lineages^17,36^ (Figure S11B). However, these studies lacked experimental evidence from mutants lacking distinct sets of sites. Our findings provide a concrete proof of this mechanism, revealing that distinct motifs within a single enhancer can indeed serve lineage-restricted functions. Specifically, E-boxes are essential for *Zeb2* expression in lymphoid progenitors and are required for the development of B cells and pDCs, and for cNK cell maturation. Meanwhile, both CEBP sites and E-boxes are utilized in myeloid progenitors, including GMPs, cMoPs, and CDPs, and are essential for normal monocyte and cDC2 development (Figure 4F). Furthermore, NFIL3 and ID2 act within a regulatory circuit that inhibits CEBP- and E protein-mediated activation of the *Zeb2* −165-kb enhancer in a subset of CDPs, enabling cDC1 specification.

This model of site-specific enhancer pleiotropy also contrasts with canonical enhancer switching^9,37–39^ (Figure S11C), in which each enhancer acts as a modular unit with activity restricted to a particular context. For example, *Irf8* expression during cDC1 development is regulated through enhancer switching. *Irf8* expression is first driven by the +56-kb enhancer (containing CEBP sites) in MPP4s and MDPs^40,41^, then by the +41-kb enhancer (containing E-boxes) in CDPs and pre-cDC1s^9,39^, and finally by the +32-kb enhancer (containing AP1-IRF composite elements) in cDC1s^39,42^. Given that different TFs are active at distinct developmental stages, each enhancer comprising the matching TF binding motifs are sequentially activated. In contrast, our findings reveal that individual binding sites within a single enhancer can independently support target gene transcription across lineages. This site-specific modularity may represent a broader architectural principle shared by other pleiotropic developmental enhancers. Notably, a recent study showed that combinations of discrete TF motifs can drive synthetic enhancer activity in multiple cell types^43^, consistent with this model.

Our results also expand the current understanding of E protein activity in hematopoiesis. establish that E-boxes within the *Zeb2* −165-kb enhancer are indispensable for normal lymphoid development, particularly of B cells, pDCs, and cNK cells. We also reveal previously underappreciated roles for E proteins in the myeloid compartment, as ΔE mice showed altered GMP and cMoP phenotypes and compromised cDC2 specification. These findings suggest a broader impact of E protein activity on hematopoiesis.

The study dissects the regulatory axis involving NFIL3, ID2, and ZEB2 in cDC1 specification, resolving a longstanding question of why ID2 is required for this lineage^15,16^. Our data suggest that NFIL3 represses *Zeb2* by antagonizing CEBP-mediated enhancer activation, while ID2 blocks E protein-driven activity at the same enhancer. We propose a multi-step model of cDC1 specification, in which NFIL3 expression in a subset of CDPs transiently reduces *Zeb2* expression, enabling induction of *Id2*. ID2, by sequestering E proteins, locks the *Zeb2* −165-kb enhancer in an “off” state (Figure S11D). Sustained ID2 expression maintains *Zeb2* repression, thereby securing the cDC1 fate. ID2 acts in two complementary ways: first, it is required, in concert with NFIL3, for the complete repression of ZEB2 (Figure 6B); and second, because NFIL3 expression is transient during cDC1 development^10^, ID2 prevents *Zeb2* reactivation after NFIL3 is downregulated, blocking redirection toward the cDC2 fate.

In the lymphoid context, we show that ZEB2 promotes B cell and pDC development in part by repressing *Id2*, consistent with prior evidence of mutual antagonism between ZEB2 and ID2 proteins^4,9,11^. While the idea that ZEB2 supports pDC development by repressing *Id2* has been proposed^11^, our study provides the first direct genetic evidence supporting this concept. Given that E2–2 is required at multiple stages of pDC development and is necessary for pDC identity maintenance^44–46^, we propose that ZEB2, whose expression is promoted by E2–2 via the −165-kb enhancer, represses *Id2* to prevent interference with E2–2-driven gene programs (Figure S11E). Analogously, ZEB2 may repress *Id2* in early B cell progenitors to enable E2A-dependent B cell specification^19–22^.

Despite these advances, several mechanistic questions remain. While NFIL3 binding to CEBP sites in the *Zeb2* −165-kb enhancer is well established, it is unclear whether NFIL3 also targets other *cis* elements within the *Zeb2* locus. Whether NFIL3 represses *Zeb2* expression solely by competing against CEBP-family activators or also recruits engaging chromatin remodelers or transcriptional co-repressors remains unknown. How ZEB2 represses *Id2* is an open question. It may involve direct binding to a *cis* element at the *Id2* locus or occur indirectly through repressing an upstream *Id2* activator. Additionally, while ZEB2 represses cDC1 development upstream of *Irf8* expression^10^, whether this involves direct repression of *Irf8* or its activators (e.g., BATF3) remains to be elucidated.

In summary, this study provides a mechanistic framework for enhancer pleiotropy based on the integration of lineage-restricted TF inputs at discrete binding sites within a shared enhancer, offering a refined model of gene regulation during development. These findings refine our understanding of gene regulation during hematopoiesis, reveal broad impacts of E-box-mediated *Zeb2* expression, and elucidate the mechanisms by which ID2 and ZEB2 regulate cDC1, B cell, and pDC development.

## RESOURCE AVAILABILITY

### Lead contact

Further information and requests for resources and reagents should be directed to and will be fulfilled by the lead contact, Professor Kenneth M. Murphy (kmurphy@wustl.edu).

### Materials availability

All unique reagents generated in this study are available from the lead contact without restriction.

### Data and code availability

The scRNA-seq dataset (lin^−^ KIT^hi-int^ BM cells) and the CUT&RUN dataset (from lin^−^ KIT^hi-int^ FLT3^+^ BM cells) generated in this study will be deposited in the Gene Expression Omnibus (GEO).

The following publicly available datasets were reanalyzed in this study: CDP ATAC-seq and H3K27ac ChIP-seq (GSE 132240 and GSE132239)^39^, pDC H3K27ac and p300 ChIP-seq (GSE66899)^42^, CEBPα, CEBPβ, and NFIL3 CUT&RUN in HoxB8 cells and NFIL3 CUT&RUN in GFP-NFIL3^+^ BM (GSE188579)^10^. E2A CUT&RUN in pro-B and pre-B cells (GSE259358)^47^, HEB ChIP-seq in MEL and CH12 cells (ENCSR349JDH and ENCSR906QEK), E2–2 ChIP-seq in CAL1 cells (GSE43876)^48^, CEBPB, E2A, and HEB ChIP-seq in K562 cells (ENCSR000EHE, ENCSR970OJY, and ENCSR189TRZ), lin^−^ KIT^int-lo^ FLT3^+^ BM scRNA-seq (GSE270060)^49^, and MDP and CDP bulk RNA-seq (GSE149761)^40^.

Any additional information required to reanalyze the data reported in this study is available upon request from the lead contact.

## METHODS

### Mice

C57BL/6 and C57BL/6-Tg(TcraTcrb)1100Mb/J (OT-I) mice were purchased from The Jackson Laboratory. OT-I mice were maintained on a CD45.1 congenic background. 129S6 mice were purchased from Taconic Biosciences. The *Zeb2* −165-kb enhancer variants ΔE2 and ΔE(2+3+4) (ΔE) were generated in-house via CRISPR/Cas9 genome editing as part of this study. The *Zeb2* −165-kb enhancer variants Δ1+2+3 (referred to as ΔC) and *Zeb2*^egfp^ −165^−/−^ (referred to as Δ−165) were generated in-house and reported in prior publications^4,10^. *Id2*^−/−^ mice were generated in-house on a 129S6 background via CRISPR/Cas9 genome editing and maintained on a mixed 129S6.C57BL/6 background for experiments. *Nfil3*^−/−^ mice were provided by A. Look and T. Mak. ZEB2-EGFP fusion protein reporter mice (*Zfhxlb*^tm2.1Yhi^) were derived from the RIKEN BioResource Center (Japan) through the National BioResource Project. *Id2*-IRES-GFP mice^50^ were provided by G. Belz.

Mice used in all experiments were on a C57BL/6 background unless otherwise specified. Experiments involving *Id2*^−/−^ mice were performed using mice on a mixed 129S6.C57BL/6 background to support viability. All mice were housed in specific pathogen-free conditions and used per protocols approved by the Animal Studies Committee of Washington University in St. Louis. Mice were maintained on 12-hour light/dark cycle at 21°C and 50% humidity. Standard chow (LabDiet 53WU Irradiated PicoLab Rodent Diet 20) was used. Mice of both sexes were used, and experimental cohorts were age- and sex-matched across genotypes, between 6 and 14 weeks of age.

### CRISPR-mediated genome editing

ΔE2, ΔE(2+3+4) (ΔE), and *Id2*^−/−^ mice were generated via CRISPR/Cas9-mediated editing. sgRNAs and the single-stranded oligodeoxynucleotide (ssODN) donor were synthesized by Integrated DNA Technologies (IDT). To generate ΔE2 mice, an sgRNA targeting site E2 (sgE2) was used. For ΔE mice, which were generated on a ΔE2 background, two sgRNAs (sgE3 and sgE4) were used along with an ssODN to facilitate HDR. To generate *Id2*^−/−^ mice, sgId2_5’ and sgId2_3’ were used.

Cas9 nuclease (IDT) was complexed with sgRNAs to form ribonucleoprotein (RNP) complexes. Day 0.5 single-cell zygotes from C57BL/6 or 129S6 mice were isolated, and RNPs were introduced via electroporation by the WUSM Department of Pathology and Immunology Transgenic Mouse Core. Electroporated zygotes were then transferred into the oviducts of pseudo-pregnant recipient mice.

Founder pups were screened by PCR using primers flanking the targeted regions. For *Zeb2* −165-kb enhancer variants, primers Mut-screen-F and Mut-screen-R (as previously described^10^) were used. ΔE involved XbaI digestion of the PCR product, which selectively cuts the mutant allele. For *Id2* genotyping, primers Id2_GT_5’, Id2_GT_Mid, and Id2_GT_3’ were used. All mutant alleles were validated by Sanger sequencing. Mice with validated mutations were outcrossed to wild-type mice, and heterozygous offspring were intercrossed to generate homozygous mice for experimental use. All oligonucleotides used for genome editing and genotyping are listed in the Key Resources Table.

### Tissue preparation for flow cytometry and cell sorting

Spleens were minced and digested in 5 mL of I10F medium with 250 μg/mL collagenase B (Roche) and 30 U/mL DNase I (Sigma) for 30 minutes at 37 °C with stirring. Following digestion, red blood cells were lysed using ACK lysis buffer. Cells were then washed with MACS buffer and passed through a 70-μm nylon mesh to obtain single-cell suspensions.

BM cells were harvested from femurs, tibias, and hips. Bones were cut open at both ends and placed vertically in a 0.5 mL microcentrifuge tube (with a hole at the bottom) nested inside a 1.5 mL tube. After adding 120 μL MACS buffer, BM was flushed by centrifugation at 5,000g for 2 minutes. Red blood cells were lysed using ACK buffer, and the resulting cells were washed with MACS buffer and passed through a-70 μm nylon mesh.

For SILP preparation, distal halves of small intestines were collected, opened longitudinally, rinsed in PBS with 2% FBS, and briefly vortexed. Tissues were incubated sequentially in 10 mL of: (1) DTT buffer (1× HBSS, 10 mM HEPES, 5 mM DTT, 2% FBS) for 20 minutes; (2) EDTA buffer (1× HBSS, 10 mM HEPES, 5 mM EDTA, 2% FBS) for 20 minutes twice; and (3) Ca^2+^/Mg^2+^ HBSS with 2% FBS and 10 mM HEPES for 10 minutes. Tissues were then minced and digested in Ca^2+^/Mg^2+^ HBSS containing 25 μg/mL Liberase (Roche), 5 μg/mL DNase I (Roche), 2% FBS, and 10 mM HEPES for 20 minutes at 37 °C with shaking (240 rpm). Digests were filtered through a 100 μm strainer, washed, and subjected to Percoll gradient centrifugation (40%/70%) at 2,000 rpm for 20 minutes at room temperature (no brake). The interphase was collected, washed with MACS buffer, and passed through a 70-μm nylon mesh to obtain single-cell suspensions

### Flow cytometry and cell sorting

Flow cytometry was performed using a FACSAria Fusion instrument (BD) and an Aurora spectral cytometer (Cytek). Data were acquired using BD FACSDiva software (v8.0) or SpectroFlo software (v2.2.0) and analyzed with FlowJo software (v10.8). Antibodies used in this study are listed in the Key Resources Table.

Single-cell suspensions were prepared from bone marrow, spleen, and SILP. Surface staining was carried out at 4 °C in magnetic-activated cell-sorting (MACS) buffer in the presence of Fc block (2.4G2). For panels involving CD16/32, FITC-conjugated anti-CD16/CD32 (2.4G2, BD) was used instead of unconjugated Fc block, at a 1:200 dilution.

Intracellular staining for E2A, CEBPα was performed using the Foxp3/Transcription Factor Staining Buffer Set (eBioscience), following the manufacturer’s protocol.

Cells were sorted on a FACSAria Fusion (BD) into Iscove’s Modified Dulbecco’s Medium supplemented with 10% FBS, 1% penicillin-streptomycin solution, 1% sodium pyruvate, 1% MEM non-essential amino acid, 1% L-glutamine solution and 55 μM β-mercaptoethanol (I10F).

### *In vitro* culture and RV enhancer reporter assays

Sequences of *Zeb2* −165-kb enhancer variants used in RV reporter assays were PCR-amplified from genomic DNA of WT or enhancer-edited mice using Phusion High-Fidelity DNA Polymerase (NEB). Amplicons were digested with ClaI and BamHI, and ligated upstream of a CMV minimal promoter driving GFP in a ClaI/BamHI-digested RV reporter vector (Thy1.1-pA-GFP-CMVp, referred to as the ‘empty’ vector), as previously described^39^. All constructs were verified by Sanger sequencing.

Retrovirus was produced by transfecting Plat-E packaging cells with the reporter constructs using TransIT-LT1 (Mirus Bio). Viral supernatants were harvested 2 days post-transfection, centrifuged to remove cellular debris, and used immediately for transduction.

BM cells were harvested from WT C57BL/6 mice, subjected to erythrocyte lysis with ACK buffer, and filtered through 70-μm nylon mesh. For DC differentiation (e.g., cDC2 and pDC), BM cells were cultured and transduced using a combined KITL/FLT3L protocol as previously described^51^. Briefly, cells were cultured in I10F medium supplemented with 5% KITL-conditioned medium and 5% FLT3L-conditioned medium for 3 days, followed by culture in I10F medium containing 5% FLT3L-conditioned medium alone for an additional 5 days. For monocyte differentiation, BM cells were cultured in I10F supplemented with 5% each of KITL-, IL-3-, and IL-6-conditioned medium for 3 days. Retroviral transduction was performed by spin infection at 729g for 1 hour at room temperature in the presence of 2 μg/mL polybrene.

### scRNA-seq

BM cells were harvested from three mice per genotype. Lineage-positive cells were depleted using antibodies against CD3ε, CD8β, CD11b, CD19, B220, Ly-6G, and TER-119. The remaining cells were stained with fluorochrome-conjugated antibodies, labeled with unique Antibody Capture TotalSeq B antibodies (BioLegend), and sorted for lin^−^ KIT^hi-int^ cells. Three samples from each genotype were pooled and resuspended in PBS containing 0.04% BSA at a final concentration of ~1,000 cells/μL. Single-cell suspensions were loaded onto a 10x Genomics Chromium Single Cell Controller at GTAC@MGI.

Library preparation, sequencing, and Cell Ranger processing were performed by GTAC@MGI. Libraries were prepared using the 10x Genomics Chromium Next GEM Single Cell 3’ Reagent Kits v3 and sequenced on an Illumina NovaSeq 6000 platform to generate 150-bp paired-end reads, targeting depths of 1 billion reads for the gene expression library and 100 million reads for the feature barcode library. Raw sequencing data were processed using Cell Ranger (v8.0.0) for alignment to the mm10 reference genome, barcode processing, UMI counting, and generation of the gene expression matrix.

### scRNA-seq data analysis

scRNA-seq downstream analysis was performed using Seurat (v5.1.0) in R (v4.3.2). Samples were demultiplexed based on feature barcodes using the HTODemux() function. After removing contaminating cells expressing lineage marker genes, unique molecular identifier counts were log-normalized using a scale factor of 10,000. The top 2,000 highly variable genes were identified and used for principal component analysis. Cell cycle phase scores were calculated, and the difference between G2M and S phase scores was regressed out. Dimensionality reduction and clustering were conducted using the FindNeighbors(), RunUMAP(), and FindClusters() functions, based on the top 32 principal components. Clustering was performed at a resolution of 0.5. Cluster annotation was conducted using SingleR (v2.4.1) with the ImmGenData reference, followed by manual curation to resolve ambiguous myeloid populations.

### CUT&RUN

CUT&RUN on sorted BM lin^−^ KIT^hi-int^ FLT3^+^ cells was performed using the CUTANA ChIC/CUT&RUN Kit (EpiCypher, Version 5), following the manufacturer’s protocol, with modifications at the MNase digestion step to reduce background, as previously described^52,53^. A total of 5 × 10^5^ cells were used per sample. Briefly, cells were immobilized on Concanavalin A-coated magnetic beads and incubated with 3 μL anti-C/EBPα antibody (8178S, Cell Signaling Technology) or 1 μL rabbit IgG negative control antibody (EpiCypher) in a total volume of 50 μL. After binding with pAG-MNase, chromatin digestion was initiated by addition of Ca^2+^ and incubated on ice for 30 minutes. Digested DNA fragments were released into the supernatant, purified using the DNA Clean & Concentrator-5 kit (ZYMO), and used for library preparation.

Libraries were generated using the NEBNext Ultra II DNA Library Prep Kit for Illumina (NEB), with modifications as described previously to preserve short DNA fragments^54^. Briefly, end-repair and A-tailing were performed using the End Prep module at 20 °C for 30 minutes followed by 50 °C for 60 minutes. Adapters (1.875 pmol) were ligated at 20 °C for 15 minutes followed by digestion with USER enzyme. Libraries were cleaned using 1.75× volumes of AMPure XP beads (Beckman Coulter) to retain short ligation products. PCR amplification was carried out for 14 cycles. Final libraries were size-selected using a two-step AMPure XP cleanup (0.8× followed by 1.2×) to enrich for fragments between 150–350 bp. Indexed libraries were pooled and sequenced as 150-bp paired-end reads on an Illumina NovaSeq X Plus platform at GTAC@MGI.

### CUT&RUN and ChIP-seq data analysis

The CUT&RUN data generated in this study from various *Zeb2* −165-kb enhancer variants were analyzed as follows. For global analysis, pair-end reads were adapter-trimmed, aligned to mm10, and deduped by GTAC@MGI using CUT&RUNTools 2.0. Raw pair-end FASTQ files were trimmed by trimmomatic with the parameters -phred33 ILLUMINACLIP:Truseq3.PE.fa:2:15:4:4:true LEADING:20 TRAILING:20 SLIDINGWINDOW:4:15 MINLEN:25. Trimmed reads were aligned to mm10 using bowtie2 with the parameters--dovetail --phred33. Aligned output was converted to BAM format and filtered using samtools view -bh -f 3 -F 4 -F 8 to retain properly paired, mapped reads where both mates are mapped. Duplicates were identified and marked by Picard Tool’s MarkDuplicates with CLEAR_DT=true ADD_PG_TAG_TO_READS=true and subsequently removed using samtools view -F 1024. Spike-in alignment, file format conversion and spike-in calibration using bedtools (v2.31.1), peak calling using SEACR (v1.3), and heatmap visualization using deepTools (v3.5.3) were performed essentially as previously described^55^.

For detailed visualization specifically at the *Zeb2* −165-kb enhancer, custom Bowtie2 indexes were generated for the relevant partial chromosome 2 regions for each *Zeb2* −165-kb enhancer variant using bowtie2-build (v2.2.5). Adapter-trimmed reads were aligned to these custom genomes using bowtie2 with the parameters --dovetail --phred33 --no-discordant --no-mixed --score-min L,0,0. Alignment output was converted to BAM, coordinate-sorted using samtools sort (v1.3.1) and indexed using samtools index. Sorted BAM files were converted to BigWig format using bamCoverage (deepTools v3.5.3) with --normalizeUsing CPM --binSize 10. Peak calling was performed using macs2 callpeaks (v2.2.9.1) with IgG control, employing parameters -g 5.5e5 -f BAMPE -q 0.1 --call-summits to generate narrowPeak files. Both BigWig and narrowPeak files were visualized using IGV (v2.16.2) on the custom WT partial chromosome 2 genome.

For publicly available datasets, raw FASTQ files were downloaded from GEO/SRA and subjected to quality control using fastqc (v0.12.1). Reads were aligned to hg38 using bowtie2 (v2.2.5) with --no-unal. Aligned reads were coordinate-sorted using samtools sort (v1.3.1), and PCR duplicates were removed using Picard Tool’s MarkDuplicates with --ASSUME_SORTED true --REMOVE_DUPLICATES true. The final BAM files were indexed using samtools index and converted to BigWig format using bam2bw from the cgpbigwig:1.6.0 Docker image. The BigWig files were hosted on the CyVerse Discovery Environment for visualization on the UCSC Genome Browser. For publicly available datasets where BigWig files were already provided on the appropriate genomes, direct links were utilized for visualization on the UCSC Genome Browser.

### Motif scanning

WT and ΔE *Zeb2* −165-kb enhancer sequences were scanned for E-box motifs using FIMO (v5.5.5). Motifs included TFE2, ITF2, and HTF4 from the HOCOMOCO Mouse v11 CORE database, as well as Tcfe2a_primary and Tcfe2a_secondary from the UniPROBE Mouse (Sci09 Cell08) database. Matches with *P* values < 10^−3^ were considered significant.

### Bulk RNA-seq reanalysis

Raw FASTQ files from publicly available datasets were downloaded from GEO/SRA. Adapter trimming was performed using trimmomatic (v0.39) with Nextera adapter sequences, followed by quality assessment with fastqc (v0.12.1). Trimmed reads were aligned to the mm10 genome using hisat2 with the parameters --dta -U within the griffithlab/rnabio:0.0.1 Docker image. Aligned reads were coordinate-sorted and indexed with samtools (v1.3.1). Resulting BAM files were converted to BigWig format using bam2bw from the cgpbigwig:1.6.0 Docker image and visualized in IGV (v2.16.2).

### In vitro cross-presentation assay

10,000 sorted splenic cDC1s or cDC2s were co-cultured with 25,000 CellTrace Violet-labeled, sorted splenic OT-I cells in the presence of 10^8^ HKLM-OVA (a gift from H. Shen) at various concentrations. Co-cultures were maintained for 3 days. T cell activation was assessed by flow cytometric analysis of CellTrace Violet dilution and CD44 upregulation.

### STAg stimulation and intracellular IL-12 detection

Splenocytes were plated at 10^6^ cells/mL and stimulated with 1 μg/mL STAg in the presence of 250 ng/mL Brefeldin A for 6 hours. Following stimulation, cells were stained for surface markers, fixed with 2% paraformaldehyde in PBS, and washed with PBS. Permeabilization and intracellular staining for IL-12 p35 and IL-12 p40 were performed in 0.5% saponin in MACS buffer at room temperature for 1 hour.

### Immunofluorescence of LCs

Epidermal sheets were prepared from mouse ear skin. Ears were split into dorsal and ventral halves, and the dorsal halves were floated epidermal side up in 1.2 U/mL in RPMI at 37 °C for 45 minutes to facilitate dermal-epidermal separation. Epidermal sheets were gently peeled off, fixed in 4% paraformaldehyde at 4 °C for 15 minutes, and washed with PBS. Sheets were blocked and stained overnight at 4 °C in 1% BSA in PBS containing antibodies against MHCII (AF594, 1:100) and EpCAM (APC, 1:100), along with DAPI nuclear counterstain. Confocal images were acquired on a TCS SP8 inverted confocal microscope.

### ELISA for IFN-α

20.000 sort-purified BM pDCs or Ly-6C^+^ monocytes (negative control) were stimulated with 6 μg/mL CpG-A 2216 (InvivoGen) for 16 hours. Cell-free supernatants were collected and diluted 1:16 in I10F prior to analysis. Diluted samples were analyzed using the Mouse IFN-α ELISA Kit (Invitrogen, BMS6027) according to the manufacturer’s instructions. Absorbance was measured on a microplate reader, and IFN-α concentrations were calculated based on a standard curve generated with recombinant IFN-α included in the kit.

## QUANTIFICATION AND STATISTICAL ANALYSIS

Statistical analyses were performed using GraphPad Prism (v10.3.1). Comparisons between two groups were conducted using unpaired, two-tailed Student’s t tests. For comparisons involving more than two groups, one-way or two-way ANOVA with with Dunnett’s post hoc test was used. For scRNA-seq data, statistical significance of gene expression differences was assessed using the Wilcoxon rank-sum test as implemented in Seurat (v5.2.1). *P* values < 0.05 were considered statistically significant. Data are presented as mean ± SD. Exact *P* values are shown in figures. Sample sizes and statistical tests are reported in figure legends.

## Supplementary Material

1

Figures S1 to S11

## Figures and Tables

**Table:** KEY RESOURCES TABLE

REAGENT or RESOURCE	SOURCE	IDENTIFIER
Antibodies		
BUV1395 rat anti-mouse CD117 (clone: 2B8)	BD Biosciences	Cat#: 564011
PE-CF594 rat anti mouse-Flt3 (clone: A2F10.1)	BD Biosciences	Cat#: 562537
PE mouse anti-rat CD90/mouse CD90.1 (clone: OX-7)	BD Biosciences	Cat#: 554898
V500 rat anti-mouse I-A/I-E (clone: M5/114.15.2)	BD Biosciences	Cat#: 562366
Alexa Fluor^®^ 700 rat anti-mouse Ly6C (clone: AL-21)	BD Biosciences	Cat#: 561237
Brilliant Violet 421 rat anti-mouse CD127 (clone:A7R34)	BD Biosciences	Cat#: 135027
Biotin anti-mouse CD19 (clone: 1D3)	BD Biosciences	Cat#: 553784
BV421 mouse anti-mouse CCR9 (clone: CW-1.2)	BD Biosciences	Cat#: 565412
APC rat anti-mouse CD43 (clone: S7)	BD Biosciences	Cat#: 560663
PE rat anti-mouse Ly-6G (clone: 1A8)	BD Biosciences	Cat#: 551461
PE rat anti-mouse Ly-6A/E (clone: D7)	BD Biosciences	Cat#: 553108
PE rat anti-mouse IgG1 (clone: A85–1)	BD Biosciences	Cat#: 550083
Purified Mouse Anti-Human E47 (clone: G127–32)	BD Biosciences	Cat#: 554077
FITC Rat Anti-Mouse CD16/CD32 (clone: 2.4G2)	BD Biosciences	Cat#: 561728
PE Mouse Anti-Mouse NK-1.1 (clone: PK136)	BD Biosciences	Cat#: 553165
PE/Cyanine7 anti-mouse CD24 (clone: M1/69)	BioLegend	Cat#: 101822
PE anti-mouse Ly-6D (clone: 49-H4)	BioLegend	Cat#: 138604
Brilliant Violet 711 anti-mouse CD115 (clone: AFS98)	BioLegend	Cat#: 135515
PE anti-mouse XCR1 (clone: ZET)	BioLegend	Cat#: 148204
Brilliant Violet 421 anti-mouse XCR1 (clone: ZET)	BioLegend	Cat#: 148216
PE anti-mouse CD45.2 (clone: 104)	BioLegend	Cat#: 109807
PE/Dazzle 594 anti-mouse/human CD45R/B220 (clone: RA3–6B2)	BioLegend	Cat#: 103258
Biotin anti-mouse TER-119 (clone: TER-119)	BioLegend	Cat#: 116204
Biotin anti-mouse Ly6G (clone: 1A8)	BioLegend	Cat#: 127604
PE anti-mouse CD226 (clone: 10E5)	BioLegend	Cat#: 128805
Brilliant Violet 605 anti-mouse CD64 (clone: X54–5/7.1)	BioLegend	Cat#: 139323
Biotin or APC anti-mouse CD3ε (clone: 145–2C11)	BioLegend	Cat#: 100311
Biotin anti-mouse/human CD45R/B220 Antibody (RA3–6B2)	BioLegend	Cat#: 103204
Brilliant Violet 785 anti-mouse/human CD45R/B220 Antibody (RA3–6B2)	BioLegend	Cat#: 103246
APC anti-mouse CD172a (SIRPα) Antibody (P84)	BioLegend	Cat#: 144014
Brilliant Violet 785 anti-mouse CX3CR1 Antibody (SA011F11)	BioLegend	Cat#: 149029
Alexa Fluor 647 anti-mouse/rat/human CD27 Antibody (LG.3A10)	BioLegend	Cat#: 124220
Biotin anti-mouse CD5 Antibody (53–7.3)	BioLegend	Cat#: 100603
Brilliant Violet 510 anti-mouse CD19 Antibody (6D5)	BioLegend	Cat#: 115546
TotalSeq-B0301 Hashtag1 Antibody	BioLegend	Cat#: 155831
TotalSeq-B0302 Hashtag2 Antibody	BioLegend	Cat#: 155833
TotalSeq-B0303 Hashtag3 Antibody	BioLegend	Cat#: 155835
C/EBPα Rabbit mAb (clone: D56F10)	Cell Signaling Technology	Cat#: 8178S
eFluor 450 rat anti-mouse CD11b (M1/70),	eBioscience	Cat#: 48-0112-82
APC anti-mouse CD317 (clone: eBio927)	eBioscience	Cat#: 17-3172-82
PerCP-eFluor 710 anti-mouse CD172a (clone: P84)	eBioscience	Cat#: 46-1721-82
PerCP-eFluor 710 anti-mouse SiglecH (clone: eBio440c)	eBioscience	Cat#: 46-0333-82
APC-eFluor 780 anti-mouse F4/80 (clone: BM8)	eBioscience	Cat#: 47-4801-82
Biotin-anti-mouse CD105 (clone: MJ7/18)	eBioscience	Cat#: 13-1051-82
V450 anti-mouse I-A/I-E (clone: M5/114.15.2)	eBioscience	Cat#: 48-5321-82
APC-eFluor 780 anti-mouse CD11c (clone: N418)	eBioscience	Cat#: 47-0114-80
PE-Cy7 anti-mouse TCRβ (clone: H57–597),	eBioscience	Cat#: 25-5961-82
PE-Cy7 anti-mouse CD25 (clone: PC61.5)	eBioscience	Cat# 25-0251-82
PerCP-eFluor 710 anti-mouse Klrg1 (clone: 2F1)	eBioscience	Cat# 46-5893-82
PE IL-12/IL-23 p40 Monoclonal Antibody (clone: C17.8)	eBioscience	Cat#: 12-7123-82
PE Rat IgG2a kappa Isotype Control (clone: eBR2a)	eBioscience	Cat#: 12-4321-81
PE ESAM Monoclonal Antibody (clone: 1G8)	eBioscience	Cat#: 12-5852-82
Fluorescein (FITC) Donkey Anti-Rabbit IgG (H+L)	Jackson ImmunoResearch	Cat#: 711-095-152
Human/Mouse IL-12/IL-35 p35 APC-conjugated Antibody (clone: 27537)	R&D Systems	Cat#: IC2191A
Mouse IgG1 APC-conjugated Antibody (clone: 11711)	R&D Systems	Cat#: IC002A
Mouse IgG1-UNLB (clone: 15H6)	SouthernBiotech	Cat#: 0102–01
Rabbit IgG-UNLB	SouthernBiotech	Cat#: 0111–01
Chemicals, Peptides, and Recombinant Proteins		
TransIT-LTI	MIRUS Bio	Cat#: MIR 2300
Iscove’s Modified Dulbecco’s Medium	GIBCO	Cat#: 12440–046
Opti-MEM Reduced Serum Medium	GIBCO	Cat#: 31985–070
Hexadimethrine bromide (Polybrene)	Sigma-Aldrich	Cat#: H9268
Fetal Bovine Serum (Characterized)	HyClone	Cat#: SH30071.03
Sodium pyruvate	Corning	Cat#: 25–000-CI
L-Glutamine	GIBCO	Cat#: 25030–164
Pen Strep (Penicillin Streptomycin)	GIBCO	Cat#: 15140–122
2-Mercaptoethanol	Sigma-Aldrich	Cat#: M3148
MEM Non-essential Amino Acid Solution (100X)	Sigma-Aldrich	Cat#: M7145
Dnase I	Sigma-Aldrich	Cat#: D4527
Collagenase B	Sigma-Aldrich	Cat#: COLLB-RO
Experimental Models: Organisms/Strains		
C57BL/6	Jackson Laboratory	JAX: 000664
DE2	This study	N/A
DE(2+3+4) (DE)	This study	N/A
D1+2+3 (DC)	Liu et al., 2022	JAX: 037704
*Zeb2*^egfp^ −165^−/−^ (D-165)	Huang et al., 2021	N/A
*Zfhxlb*^tm2.1Yhi^ (ZEB2-EGFP)	Riken	N/A
129S6	Taconic Biosciences	129SVE-F; 129SVE-M
*Id2* ^−/−^	This study	N/A
*Id2*-IRES-GFP	G. Belz	N/A
Oligonucleotides		
sgE2: TGATTGCACACACCTGTTTG	This study	N/A
sgE3: GTTAACTGCTCAAGCAGCTG	This study	N/A
sgE4: GATAACGTTCTTGAAGCATA	This study	N/A
ssODN: CCCCAAAAAGGGGAAACTGAACTCACTGCTCCTCCTGAGCTGTGCAAAATAGCAGAAAAtctagaCTGCTTGAGCAGTTAACAGATATCAGTAAAATACCCATGTTACATAATTAAGGATAACGTTCTTGAAGggatcctGCTGAAGGTATTATACTCTCATTAAAACTTTAGGTAGCAAACAATTTAGCACATTCTCAT	This study	N/A
sgId2_5’: GACGGGAGCCCTTCCACCAA	This study	N/A
sgId2_3’: TACCGAAACGCGCACACAGG	This study	N/A
Mut-screen-F: GAAGAGGGCAATACCTTCCA	Liu et al., 2022	N/A
Mut-screen-R: CGAGGCAACCACATAACCTT	Liu et al., 2022	N/A
Id2_GT_5’: CACAATTGCACACTCAGGCC	This study	N/A
Id2_GT_Mid: CGGGGCGTCTTTTATGTGC	This study	N/A
Id2_GT_3’: TGTAGTCCTGAGCAACCACA	This study	N/A
