# Supplementary material for: ID2 secures cDC1 specification by antagonizing E proteins at a pleiotropic Zeb2 enhancer
Source: Res Sq. 2025 Sep 4:rs.3.rs-7455813. Preprint. [Version 1] doi: 10.21203/rs.3.rs-7455813/v1 (PMC12425045; doi:10.21203/rs.3.rs-7455813/v1)
Supplement: 1 [file NIHPPRS7455813V1-supplement-1.pdf]

Figure S1, related to Figure 1

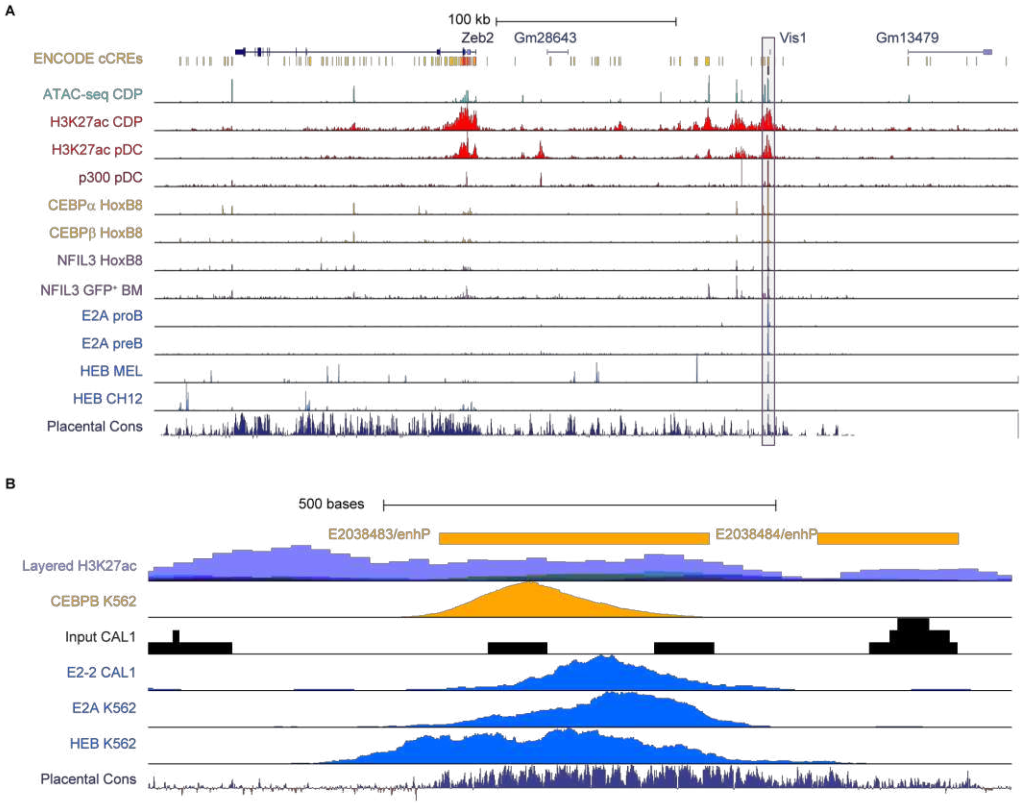

Figure S1. Both CEBPs and E proteins bind the *Zeb2* -165-kb enhancer and its human ortholog (related to Figure 1).

(A) Genomic features across the *Zeb2* locus (chr2:44,938,455-45,418,454; mm10) with the -165-kb enhancer highlighted. ENCODE cCREs, ATAC-seq, ChIP-seq, CUT&RUN, and Placental Cons tracks are displayed for the indicated targets and cell types. Y-axes are auto-scaled.

(B) Genomic features surrounding the human ortholog of the *Zeb2* -165-kb enhancer (chr2:144,703,552-144,704,651; hg38). ENCODE cCREs, ChIP-seq, CUT&RUN, and Placental Cons tracks are displayed for the indicated targets and cell lines. Y-axes are auto-scaled.

Figure S2, related to Figure 1

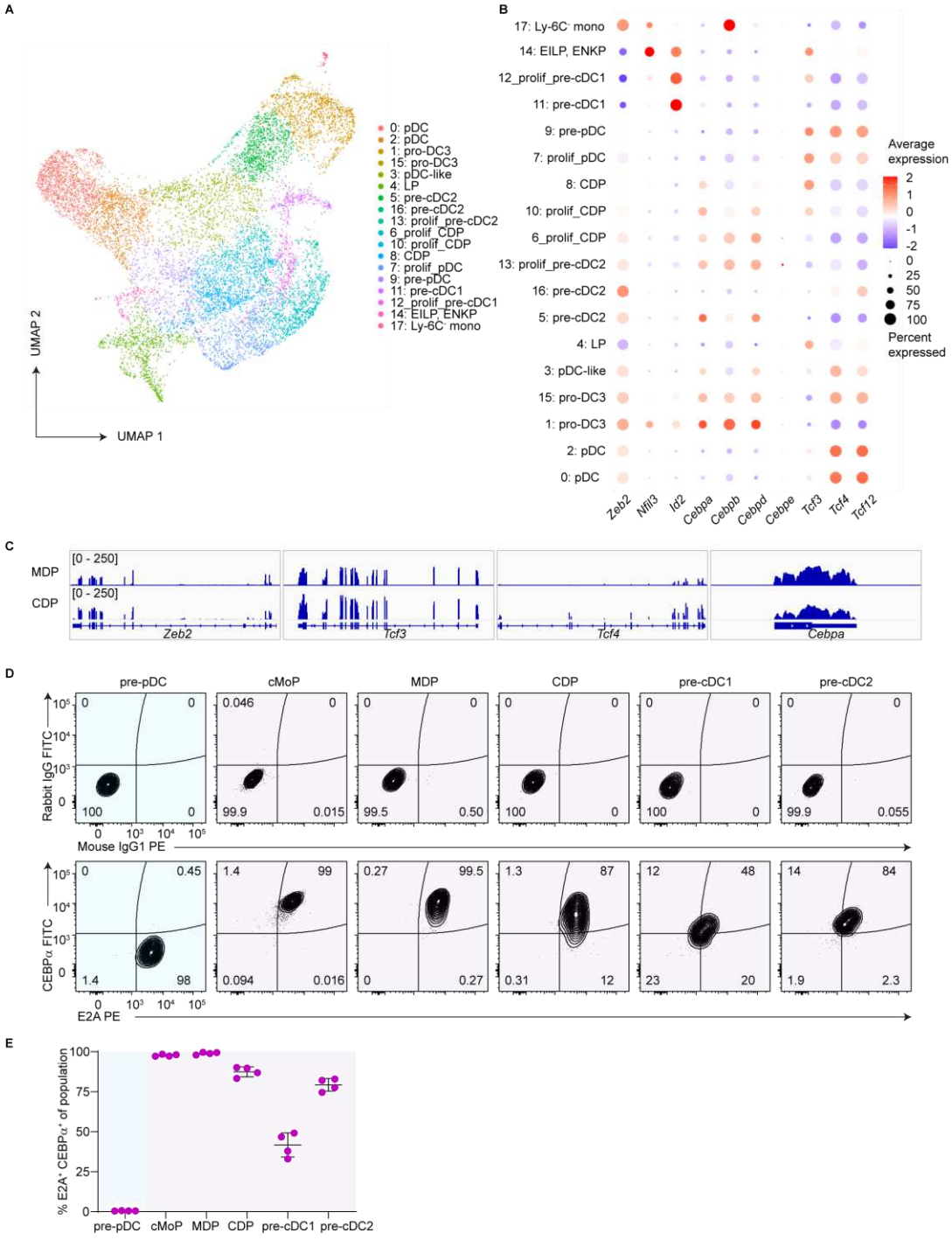

990 **Figure S2. Both CEBPs and E proteins are expressed in DC progenitors (related to Figure**
991 **1).**

(A-B) scRNA-seq data from GSE270060, derived from BM lin^{-} KIT$^{int\text{-}lo}$ FLT3^{+} cells, were reanalyzed to extract WT cells and visualize expressions of the indicated genes.
(A) UMAP showing annotated cell populations.
(B) Dot plot showing expression of selected TF genes across annotated cell populations.
(C) Bulk RNA-seq tracks of the *Zeb2*, *Tcf3*, *Tcf4*, and *Cebpa* loci in MDPs and CDPs.
(D and E) Intracellular staining of WT C57BL/6 BM using isotype control or using E2A and CEBPα antibodies (n = 4 mice; two independent experiments).
(D) Representative flow cytometry plots showing isotype control (top) and E2A/CEBPα (bottom) staining in the indicated populations.
(E) Frequencies of E2A^{+} CEBPα^{+} cells within the indicated populations (mean ± SD).

Figure S3, related to Figure 2

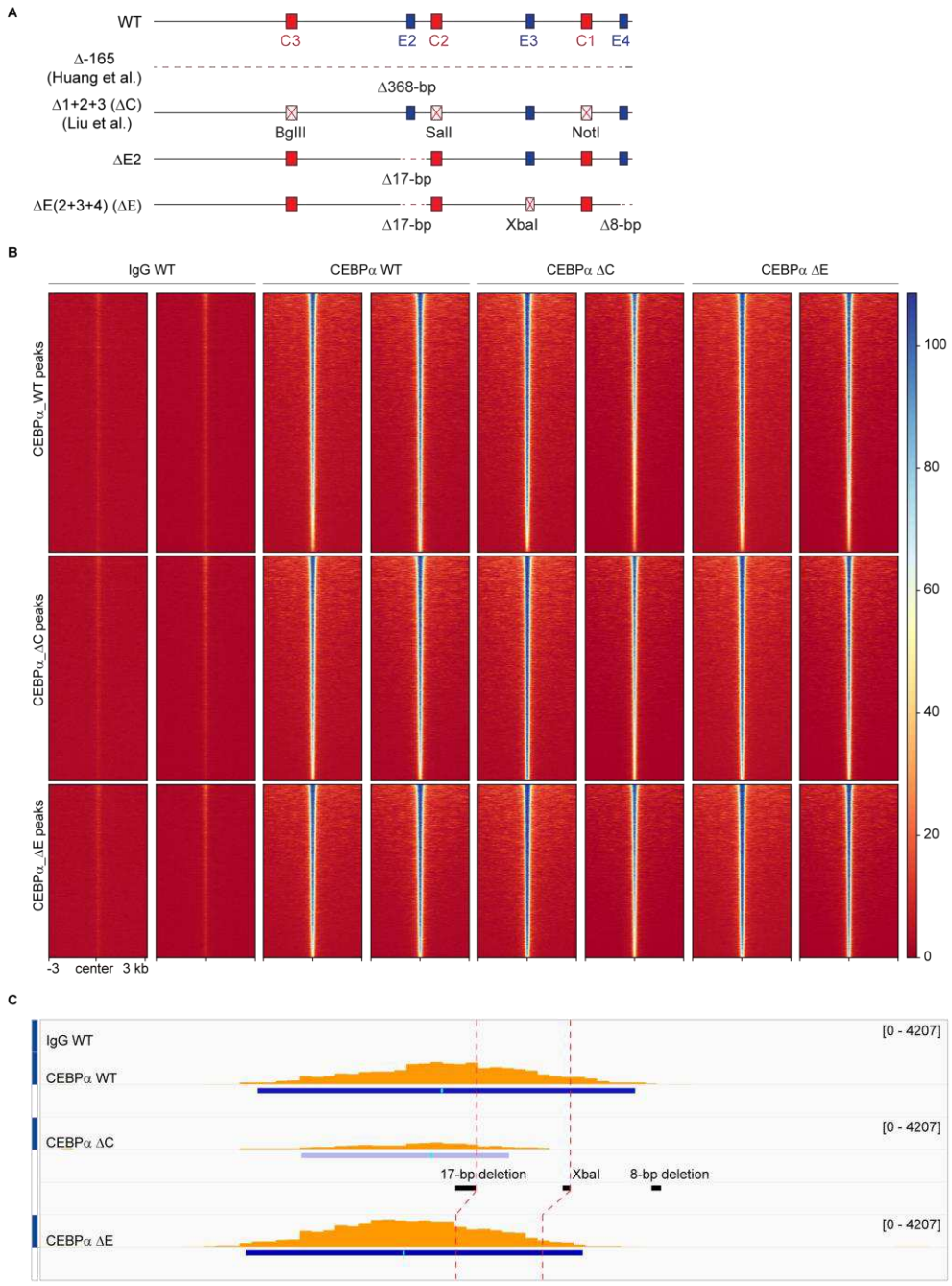

1003 **Figure S3. The ΔE variant of the *Zeb2* -165-kb enhancer retains CEBPα binding (related to**

1004 **Figure 2).**

1005 (A) Schematic illustrating *Zeb2* -165-kb enhancer variants used in this study.

(B and C) CUT&RUN for IgG (control) and CEBPα on BM lin^{-} KIT$^{hi\text{-}int}$ FLT3^{+} cells sort-purified from mice with the indicated genotypes (n =2; two independent experiments). Lineage markers used for depletion included CD3ε, CD11b, CD11c, CD19, B220, CD105, IL-7Rα, Ly-6G, TER-119, NK1.1, and MHCII.

(B) Heatmap showing CUT&RUN signal intensity across all identified CEBPα peaks for each sample.

(C) CEBPα binding at the *Zeb2* -165-kb enhancer. For precise visualization, reads were aligned to a custom partial chromosome 2 specific to each genotype. Shown is data from one representative experiment.

Figure S4, related to Figures 2 and 3

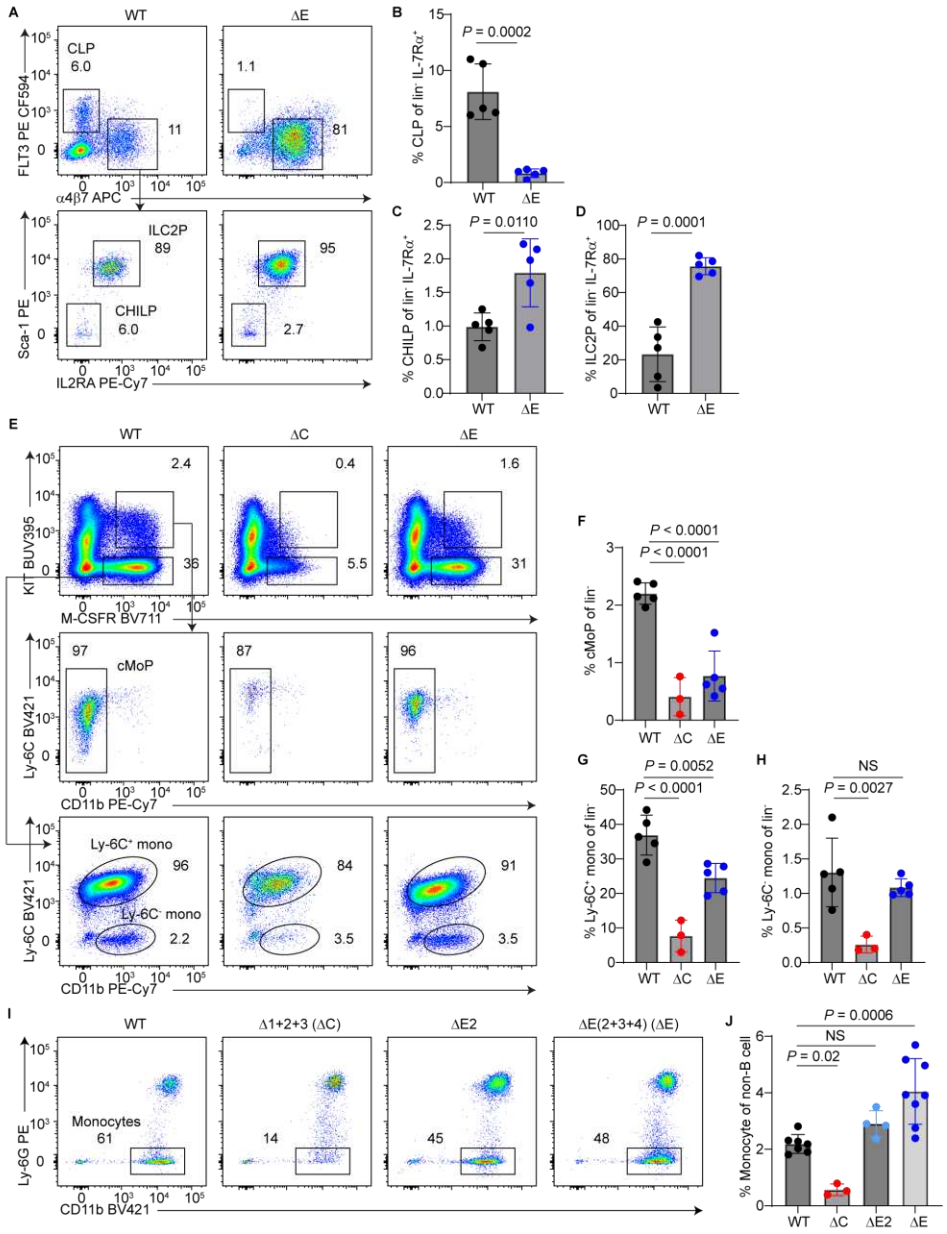

1016 **Figure S4. E-boxes within the *Zeb2* -165-kb enhancer are required for balanced**

1017 **lymphopoiesis (related to figures 2 and 3).**

1018 (A-D) Flow cytometric analysis of BM from WT and ΔE mice for the indicated lymphoid

1019 progenitor populations (n = 5 mice; three independent experiments).

(A) Representative plots showing gating of CLPs, CHILPs, and ILC2Ps. Pre-gate: lin$^-$ IL-7Rα^+ cells. Lineage markers included CD3ε, CD4, CD5, CD8β, CD11b, CD11c, CD19, Gr-1, Ly-6G, TER-119, and NK1.1.

(B) Frequencies of CLPs among lin$^-$ IL-7Rα^+ cells.

(C) Frequencies of CHILPs among lin$^-$ IL-7Rα^+ cells.

(D) Frequencies of ILC2Ps among lin$^-$ IL-7Rα^+ cells.

(E-H) Flow cytometric analysis of BM from mice with the indicated *Zeb2* -165-kb enhancer genotypes for monocyte-related populations (n = 3-5 mice; four independent experiments).

(E) Representative plots showing gating of cMoPs, Ly-6C$^+$ monocytes (Ly-6C$^+$ mono), and Ly-6C$^-$ monocytes (Ly-6C$^-$ mono). Pre-gate: lin$^-$ FLT3$^-$ cells. Lineage markers included CD3ε, CD19, B220, CD105, IL-7Rα, Ly-6G, and TER-119.

(F) Frequencies of cMoPs among lin$^-$ cells.

(G) Frequencies of Ly-6C$^+$ monocytes among lin$^-$ cells.

(H) Frequencies of Ly-6C$^-$ monocytes among lin$^-$ cells.

(I and J) Flow cytometric analysis of splenic monocytes from mice with the indicated *Zeb2* -165-kb enhancer genotypes (n = 3-8 mice; four independent experiments).

(I) Representative plots showing monocyte gating. Pre-gate: KIT$^-$ M-CSFR$^+$ MHCII$^-$ cells.

(J) Frequencies of monocytes among non-B cells. B cells were gated as B220$^+$ CD19$^+$ cells as in Figure 2B.

Bar graphs represent mean ± SD. Statistical significance was assessed using one-way ANOVA or unpaired two-tailed t tests, as appropriate; NS, not significant.

Figure S5, related to Figure 3

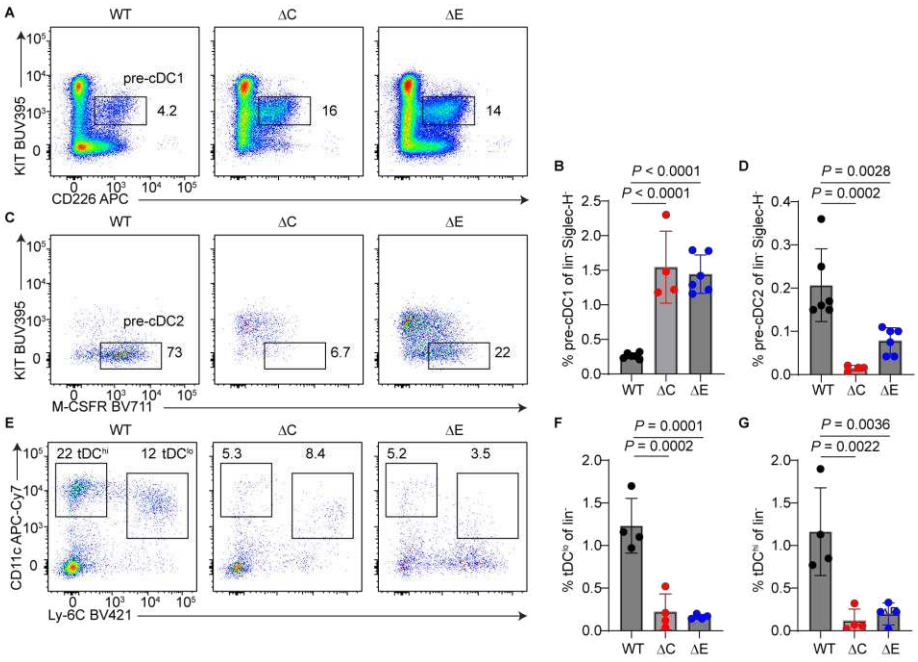

Figure S5. Both CEBP sites and E-boxes within the *Zeb2* -165-kb enhancer regulate enhancer activity and cDC subset differentiation (related to figure 3).

(A-D) Flow cytometric analysis of BM from mice with the indicated *Zeb2* -165-kb enhancer genotypes for pre-cDC1 and pre-cDC2 populations (n = 4-6 mice; five independent experiments).

(A) Representative plots showing pre-cDC1 gating. Pre-gate: lin$^-$ Siglec-H$^-$ FLT3$^+$ cells. Lineage markers included CD3ε, CD19, B220, CD105, IL-7Rα, Ly-6G, and TER-119.

(B) Frequencies of pre-cDC1s among lin$^-$ Siglec-H$^-$ cells.

(C) Representative plots showing pre-cDC2 gating. Pre-gate: lin$^-$ Siglec-H$^-$ FLT3$^+$ CD11c$^-$ MHCII$^-$ cells. Lineage markers as in (A).

(D) Frequencies of pre-cDC2s among lin$^-$ Siglec-H$^-$ cells.

(E-G) Flow cytometric analysis of splenic tDC subsets from mice with the indicated *Zeb2* -165-kb enhancer genotypes (n = 4 mice; four independent experiments).

(E) Representative plots showing tDC^{lo} and tDC^{hi} gating. Pre-gate: lin^- $XCR1^-$ $CD11b^-$ $CX3CR1^+$ cells. Lineage markers included CD3ε, CD19, Ly-6G, and NK1.1.

(F) Frequencies of tDC^{lo} among lin^- cells.

(G) Frequencies of tDC^{hi} among lin^- cells.

Bar graphs represent mean ± SD. Statistical significance was assessed using one-way ANOVA; NS, not significant.

Figure S6, related to Figure 4

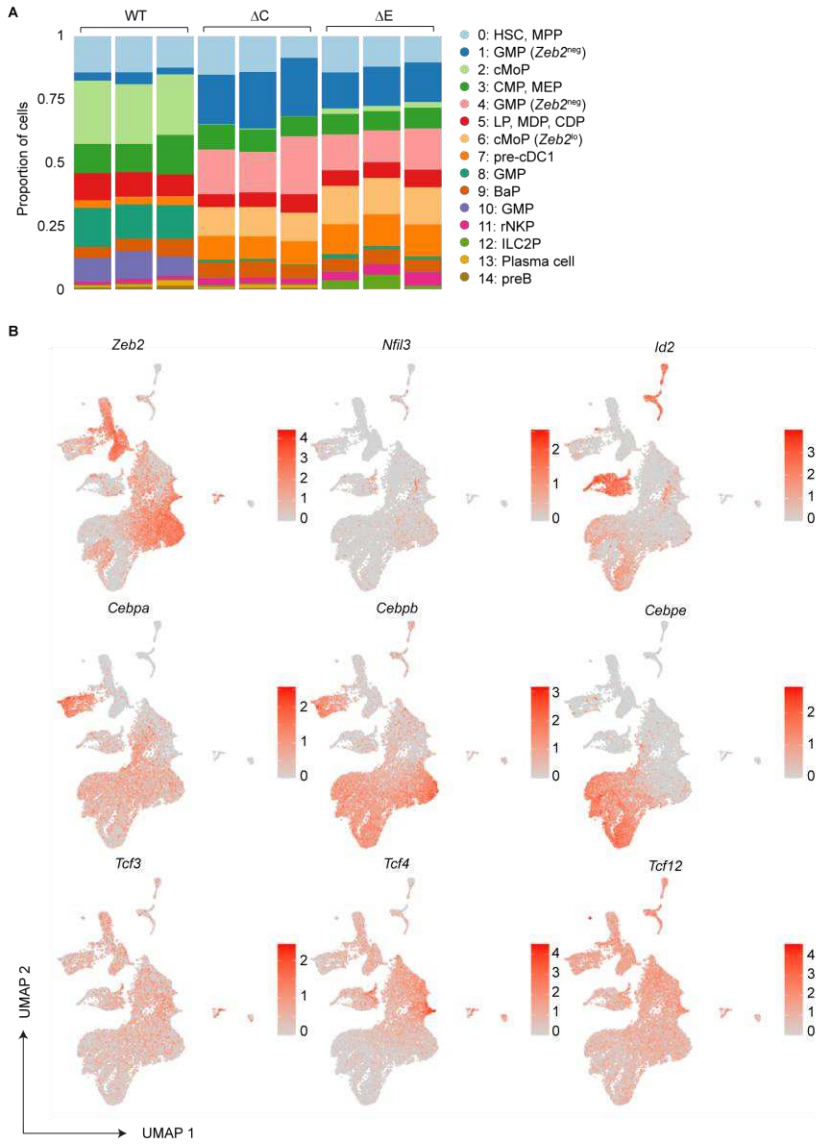

Figure S6. Normal hematopoiesis requires both CEBP sites and E-boxes in the *Zeb2* -165-kb enhancer (related to Figure 4).

(A) Stacked bar plot showing the proportions of each identified cell type across individual mice from the scRNA-seq experiment in Figure 4.

(B) Expression of selected TF genes projected onto the UMAP shown in Figure 4.

Figure S7, related to Figure 5

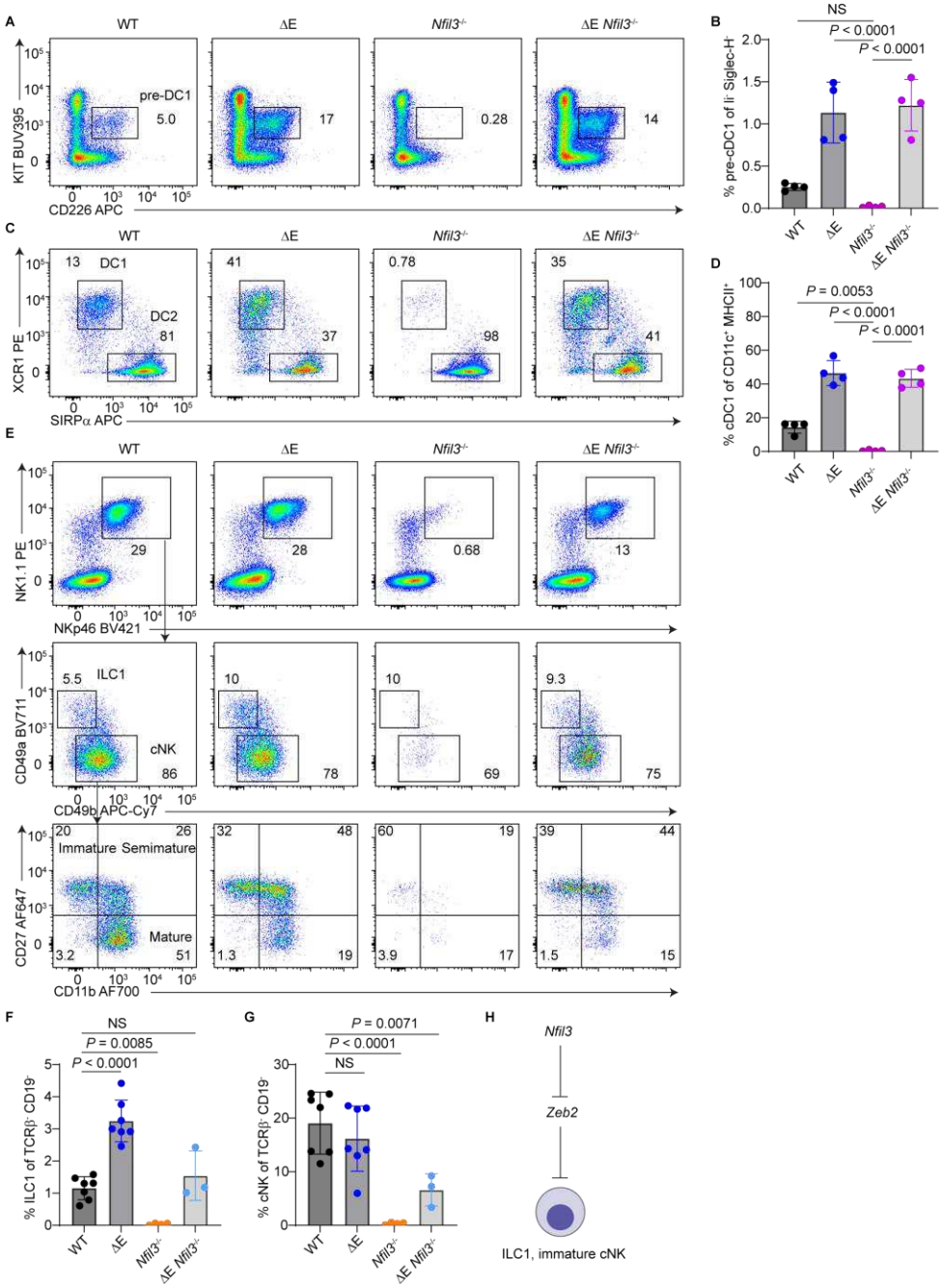

Figure S7 NFIL3 promotes cDC1, ILC1, and immature cNK cell development by repressing *Zeb2* (related to Figure 5).

(A and B) Flow cytometric analysis of BM pre-cDC1s from mice with the indicated genotypes (n = 4 mice; four independent experiments).

(A) Representative plots showing pre-cDC1 gating. Pre-gate: lin^- $Siglec\text{-}H^-$ $FLT3^+$ cells. Lineage markers included CD3ε, CD19, B220, CD105, IL-7Rα, Ly-6G, and TER-119.

(B) Frequencies of pre-cDC1s among lin^- $Siglec\text{-}H^-$ cells.

(C and D) Flow cytometric analysis of splenic cDCs from mice with the indicated genotypes (n = 4 mice; four independent experiments).

(C) Representative plots showing cDC1 and cDC2 populations. Pre-gate: $B220^-$ $Siglec\text{-}H^-$ $CD11c^+$ $MHCII^+$ cells (cDCs).

(D) Frequencies of cDC1s among cDCs.

(E-G) Flow cytometric analysis of splenic ILCs from mice with the indicated genotypes (n = 3-7 mice; six independent experiments).

(E) Representative plots showing gating of ILC1s, total cNK cells, and cNK maturation stages. Pre-gate: $TCR\beta^-$ $CD19^-$ cells.

(F) Frequencies of ILC1s among $TCR\beta^-$ $CD19^-$ cells. WT and ΔE data points were also presented in Figure 2G.

(G) Frequencies of cNK cells among $TCR\beta^-$ $CD19^-$ cells. The WT and ΔE data points were also presented in Figure 2H.

(H) Schematic summarizing the epistatic relationship between *Nfil3* and *Zeb2* in ILC1 and immature cNK cell development.

Bar graphs represent mean ± SD. Statistical significance was assessed using one-way ANOVA; NS, not significant.

Figure S8, related to Figure 6

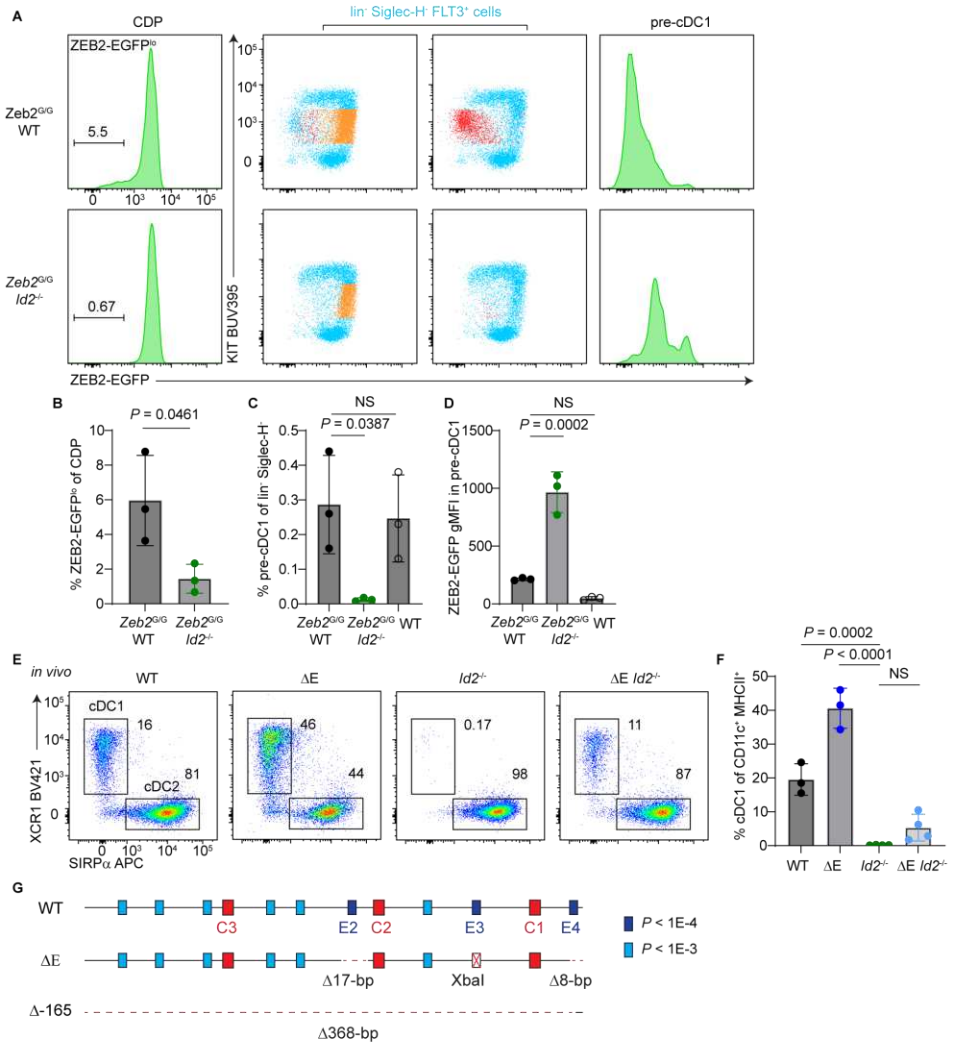

1093 **Figure S8. ID2 promotes cDC1 development by inhibiting E protein activity at the *Zeb2* -**
1094 **165-kb enhancer (related to Figure 6).**

1095 (A-D) Flow cytometric analysis of ZEB2-EGFP expression in BM from mice with the indicated
1096 genotypes (n = 3 mice; three independent experiments).

1097 (A) Representative plots highlighting ZEB2-EGFP expression in the indicated populations.
1098 CDPs were gated as lin$^-$ Siglec-H$^-$ KIT^{int} FLT3$^+$ M-CSFR$^+$ CD11c$^-$ MHCII$^-$; pre-cDC1s were
1099 gated as lin$^-$ Siglec-H$^-$ KIT^{int} FLT3$^+$ CD226$^+$ cells. Lineage markers included CD3ε, CD11b,
1100 CD19, B220, CD105, IL-7Rα, Ly-6G, and TER-119.

(B) Frequencies of ZEB2-EGFP^{lo} CDPs among total CDPs.

(C) Frequencies of pre-cDC1s among lin$^-$ Siglec-H$^-$ cells.

(D) ZEB2-EGFP gMFI in pre-cDC1s.

(E and F) Flow cytometric analysis of spleens from mice with the indicated genotypes (n = 3-4 mice; four independent experiments).

(E) Representative plots showing cDC1 and cDC2 gating. Pre-gate: B220$^-$ Siglec-H$^-$ CD11c$^+$ MHCII$^+$ cells (cDCs).

(F) Frequencies of cDC1s among cDCs.

(G) Schematic showing *Zeb2* -165-kb enhancer variants showing high-confidence E-boxes (E2, E3, and E4; dark blue) and additional low-confidence E-boxes (light blue) identified using FIMO motif scanning. WT and ΔE sequences were analyzed with E-box motifs from HOCOMOCO Mouse (v11 CORE) and UniPROBE Mouse (Sci09 Cell08) databases.

Bar graphs represent mean ± SD. Statistical significance was assessed using one-way ANOVA or unpaired two-tailed t tests, as appropriate; NS, not significant.

Figure S9, related to Figure 6

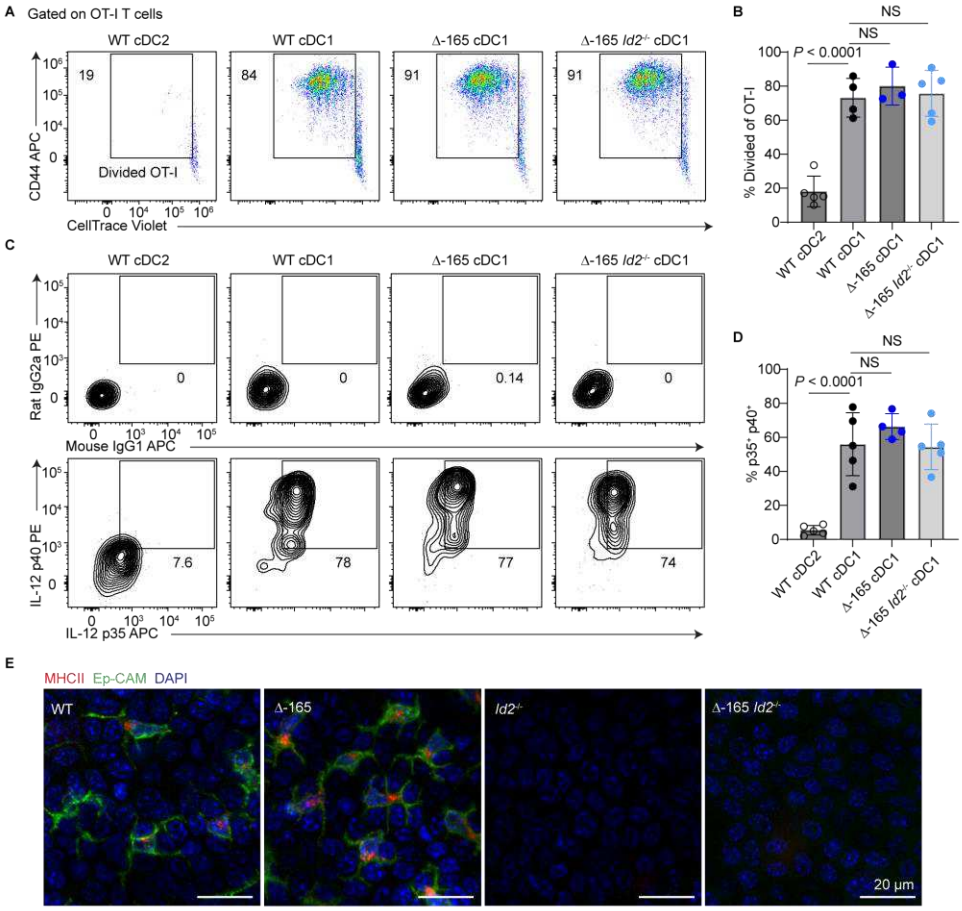

1116 **Figure S9. ID2 is dispensable for cDC1 functions but required for LC development (related**
1117 **to Figure 6).**

1118 (A and B) Splenic cDC1s or cDC2s (negative control) were sort-purified from mice with the
1119 indicated genotypes and assessed for their capacity to cross-present HKLM-OVA to native OT-I
1120 cells *in vitro* (n = 3-5 mice; four independent experiments).

1121 (A) Representative flow cytometry plots showing OT-I cells co-cultured with the indicated
1122 antigen-presenting cells, showing CTV dilution and CD44 expression. Pre-gate: $CD45.1^+$
1123 $CD45.2^-$ $CD8\alpha^+$ $Va2^+$ (OT-I) cells.

1124 (B) Frequencies of divided OT-I cells.

(C and D) Intracellular cytokine staining for IL-12 production following *Toxoplasma gondii* STAg stimulation. Splenocytes from mice with the indicated genotypes were stimulated for 6 hours in the presence of Brefeldin A (n = 4–5 mice; four independent experiments).
(C) Representative plots showing isotype control (top) and IL-12 p35/p40 staining (bottom) in cDC1s and cDC2s (negative control). cDC1s were gated as $B220^-$ $Ly\text{-}6C^-$ $CD11c^+$ $MHCII^+$ $XCR1^+$ $SIRP\alpha^-$; cDC2s were gated as $B220^-$ $Ly\text{-}6C^-$ $CD11c^+$ $MHCII^+$ $XCR1^-$ $SIRPa^+$ cells.
(D) Frequencies of $p35^+$ $p40^+$ cells.
(E) Immunofluorescence images highlighting epidermal LCs identified as $MHCII^+$ $Ep\text{-}CAM^+$ cells in ear epidermal sheets from mice with the indicated genotypes (representative of n = 3; three independent experiments).
Bar graphs represent mean ± SD. Statistical significance was assessed using one-way ANOVA; NS, not significant.

Figure S10, related to Figure 7

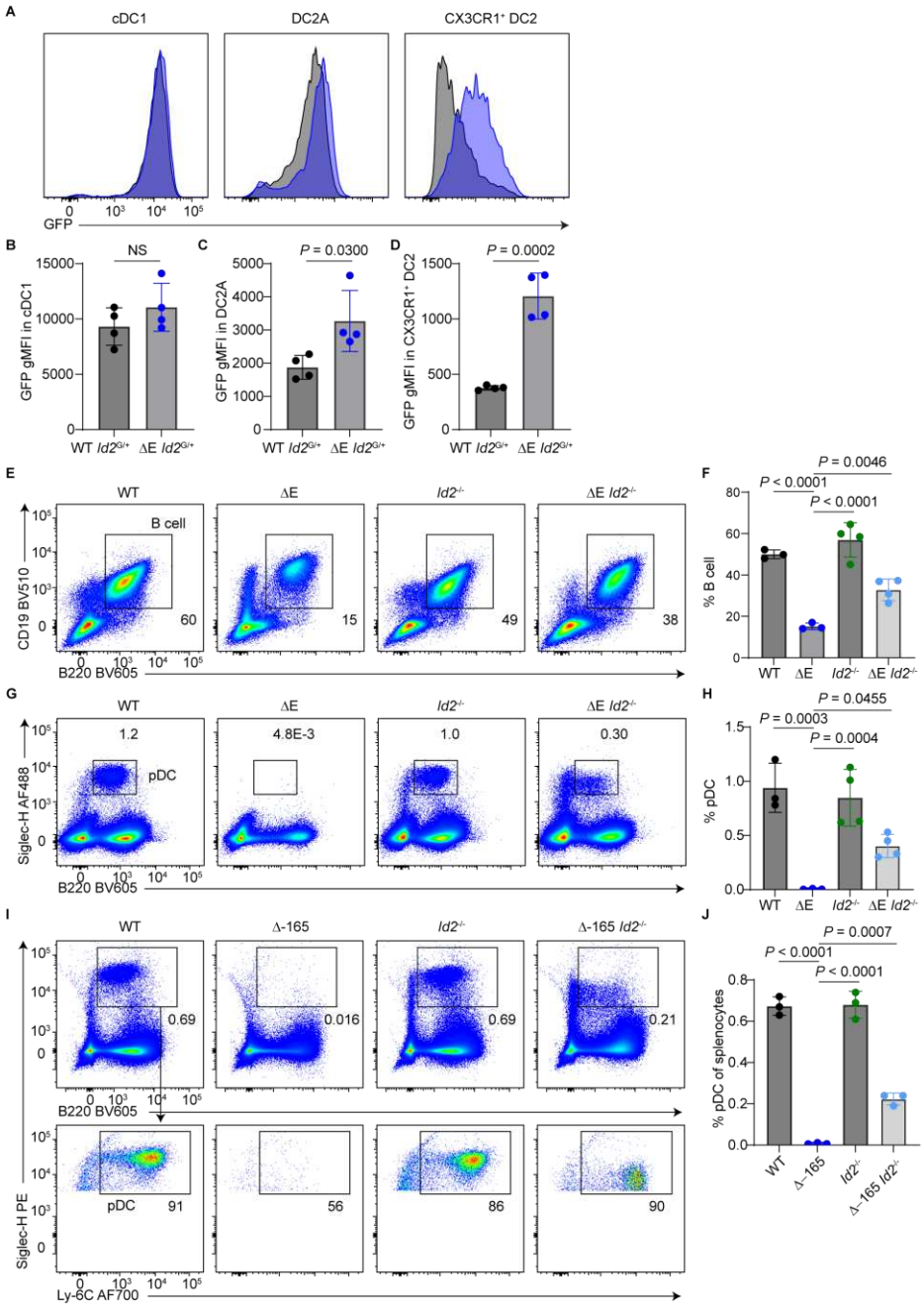

1138 **Figure S10. *Id2* is epistatic to *Zeb2* in regulating B cell, pDC, and ILC2 development**

1139 **(related to Figure 7).**

1140 (A-D) Flow cytometric analysis of *Id2*-GFP expression in splenic cDC subsets from WT and ΔE

1141 mice carrying an *Id2*-IRES-GFP reporter (n = 4 mice; three independent experiments). cDC1s

were gated as B220^{-} CD11c^{+} MHCII^{+} CD24^{+} SIRPα^{-}; DC2As as B220^{-} CD11c^{+} MHCII^{+} SIRPα^{+} ESAM^{+}; CX3CR1^{+} DC2s (including DC2Bs and DC3s) as B220^{-} CD11c^{+} MHCII^{+} SIRPα^{+} CX3CR1^{+} cells.

(A) Representative histograms showing GFP expression.

(B) GFP gMFI in cDC1s.

(C) GFP gMFI in DC2As.

(D) GFP gMFI in CX3CR1^{+} DC2s.

(E-J) Flow cytometric analysis of spleens from mice with the indicated genotypes.

(E) Representative plots showing B cell gating.

(F) Frequencies of B cells among splenocytes (n = 3-4 mice; four independent experiments).

(G) Representative plots showing pDC gating.

(H) Frequencies of pDCs among splenocytes (n = 3-4 mice; four independent experiments).

(I) Representative plots showing splenic pDC gating.

(J) Frequencies of pDC among splenocytes (n = 3 mice; three independent experiments).

Bar graphs represent mean ± SD. Statistical significance was assessed using one-way ANOVA; NS, not significant.

Figure S11

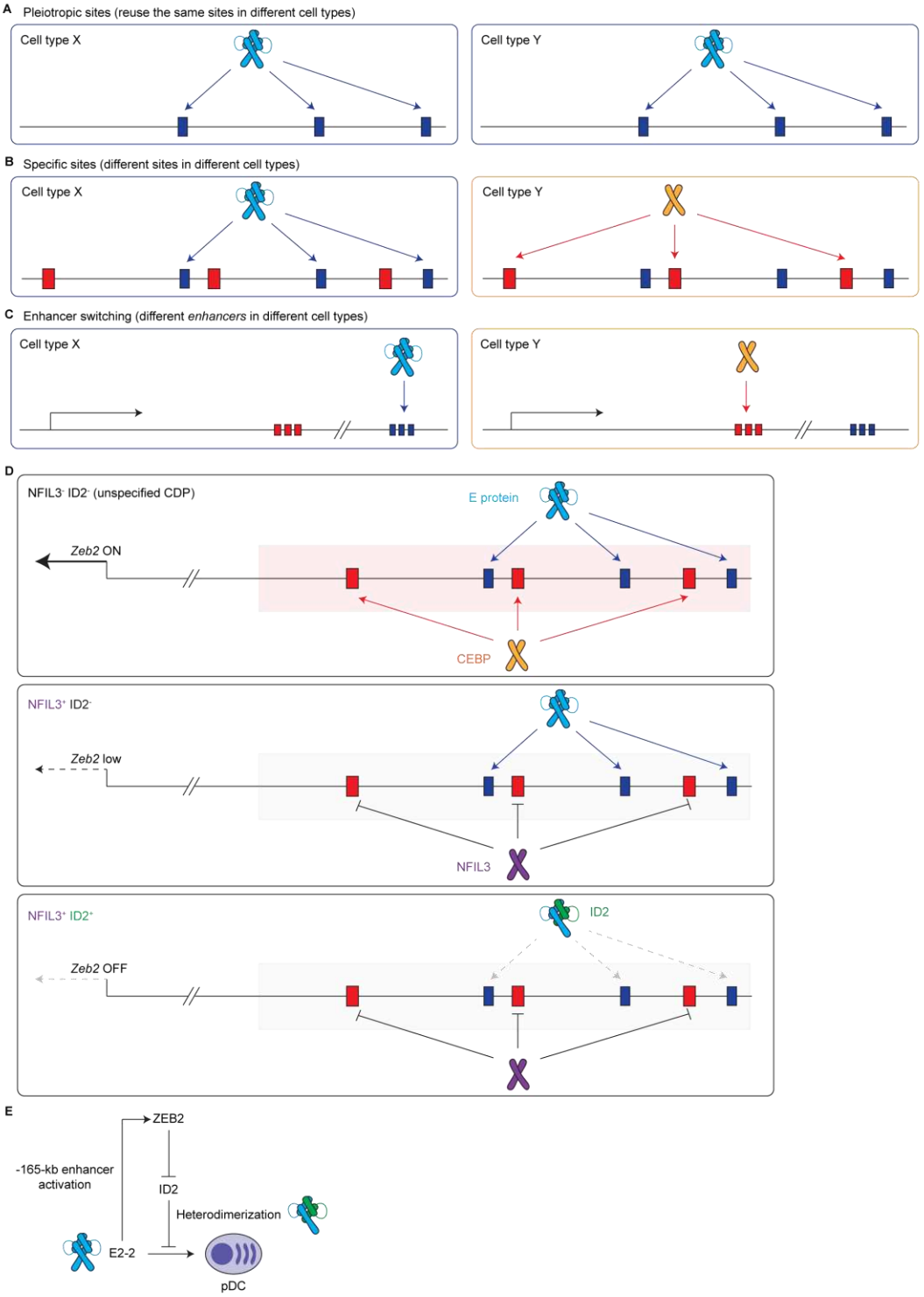

1159 **Figure S11. Schematic summary of enhancer pleiotropy and transcriptional control of**

1160 ***Zeb2*.**

(A and B) Two distinct mechanisms of enhancer pleiotropy.

(A) Site pleiotropy: the same TFBSs are reused in multiple cell types (e.g., cell types X and Y).

(B) Site-specific pleiotropy: distinct TFBSs within a single enhancer are utilized in cell types X and Y, depending on the TFs expressed in each cell type.

(C) Enhancer switching model: distinct enhancers are activated in cell types X and Y, due to differences in TF expression patterns.

(D) Working model illustrating how NFIL3 and ID2 repress *Zeb2* expression by sequentially displacing CEBPs and E proteins from the *Zeb2* -165-kb enhancer.

(E) Working model illustrating how ZEB2 promotes pDC development by repressing ID2.
